# Evolutionary History of Wild Barley (*Hordeum vulgare* subsp. *spontaneum*) Analyzed Using Multilocus Sequence Data and Paleodistribution Modeling

**DOI:** 10.1093/gbe/evu047

**Published:** 2014-02-27

**Authors:** Sabine S. Jakob, Dennis Rödder, Jan O. Engler, Salar Shaaf, Hakan Özkan, Frank R. Blattner, Benjamin Kilian

**Affiliations:** ^1^Leibniz Institute of Plant Genetics and Crop Research (IPK), Gatersleben, Germany; ^2^Zoologisches Forschungsmuseum Alexander Koenig, Bonn, Germany; ^3^Department of Wildlife Sciences, University of Göttingen, Germany; ^4^Department of Agronomy and Plant Breeding, College of Agriculture and Natural Resources, Islamic Azad University, Sanandaj branch, Sanandaj, Iran; ^5^Department of Field Crops, Faculty of Agriculture, University of Çukurova, Adana, Turkey; ^6^German Centre for Integrative Biodiversity Research (iDiv) Halle-Jena-Leipzig, Leipzig, Germany

**Keywords:** phylogeography, genetic diversity, population genetics, species distribution models, population structure, domestication

## Abstract

Studies of *Hordeum vulgare* subsp. *spontaneum*, the wild progenitor of cultivated barley, have mostly relied on materials collected decades ago and maintained since then ex situ in germplasm repositories. We analyzed spatial genetic variation in wild barley populations collected rather recently, exploring sequence variations at seven single-copy nuclear loci, and inferred the relationships among these populations and toward the genepool of the crop. The wild barley collection covers the whole natural distribution area from the Mediterranean to Middle Asia. In contrast to earlier studies, Bayesian assignment analyses revealed three population clusters, in the Levant, Turkey, and east of Turkey, respectively. Genetic diversity was exceptionally high in the Levant, while eastern populations were depleted of private alleles. Species distribution modeling based on climate parameters and extant occurrence points of the taxon inferred suitable habitat conditions during the ice-age, particularly in the Levant and Turkey. Together with the ecologically wide range of habitats, they might contribute to structured but long-term stable populations in this region and their high genetic diversity. For recently collected individuals, Bayesian assignment to geographic clusters was generally unambiguous, but materials from genebanks often showed accessions that were not placed according to their assumed geographic origin or showed traces of introgression from cultivated barley. We assign this to gene flow among accessions during ex situ maintenance. Evolutionary studies based on such materials might therefore result in wrong conclusions regarding the history of the species or the origin and mode of domestication of the crop, depending on the accessions included.

## Introduction

Barley (*Hordeum vulgare* subsp. *vulgare*) is one of the major cereals worldwide and is among the oldest domesticated crops. Barley was selected from its wild progenitor *H. vulgare* subsp. *spontaneum*, which is considered an important source for barley improvement under changing climatic conditions (Bothmer et al. 1995; Nevo et al. 2012). Both taxa are diploid (2*n* = 14), predominantly self-pollinated, and fully interfertile (Zohary and Hopf 2000). Wild and domesticated barley differ in several phenotypic characteristics, collectively referred to as the domestication syndrome (Hammer 1984).

Wild barley naturally occurs in Southwest Asia, from the eastern Mediterranean coasts to the semideserts of Afghanistan (Schiemann 1948; Harlan and Zohary 1966). The taxon constitutes an important annual element of open herbaceous and park-like vegetation in the Fertile Crescent. Outside of this region, it is mainly restricted to artificial (secondary) habitats and occurs particularly in the east of its distribution area only in widely scattered populations. Long barbed lemma awns, together with the disintegration of the ear after ripening that releases the arrow-like spikelets, provide excellent adaptation to epizoochory and colonization. It is unclear whether populations of wild barley in Morocco, Ethiopia, and Tibet are native, introduced due to human activities, or represent feral forms of cultivated barley (Bjørnstad et al. 1997; Badr et al. 2000; Molina-Cano et al. 2005).

During the last century, wild and domesticated barley was collected all over its distribution area and seed samples were stored and maintained in ex situ genebanks. Barley is maintained in more than 200 collections worldwide, amounting to ∼470,000 accessions (Knüpffer 2009). This stock of accessions forms the major source of plant materials included in studies of different aspects of genetic diversity in barley (Badr et al. 2000; Morrell and Clegg 2007; Hofinger et al. 2011; Russell et al. 2011; Hübner et al. 2012; Kilian and Graner 2012; Pasam et al. 2012). Only a few studies have explored natural populations of subsp. *spontaneum* (Özkan et al. 2005; Hübner et al. 2009), that is, using materials collected directly from the wild without long-term ex situ storage. These studies focused, however, on narrow geographic areas and not on the entire natural distribution range of the taxon. In spite of the large number of studies dealing with subsp*. vulgare*, detailed information on the evolutionary history and extant population structure of subsp. *spontaneum* within its natural distribution range is still lacking.

In this study, we describe phylogeographic and population genetic analyses of spatial genetic variation in wild barley populations based on seven nuclear loci and provide a first analysis mainly based on population samples freshly collected throughout the whole natural distribution range. Most importantly, we included samples from Turkey, an area not adequately covered in earlier studies. Ex situ-propagated wild barley accessions from genebank repositories were included for comparison and to close collection gaps. The collection was complemented by a diverse set of domesticated genotypes to compare the allelic composition of the crop with that of its wild progenitor. We combined phylogeographic data with climate-based modeling of wild barley distribution during the last 21,000 years (Peterson et al. 1999; Pearman et al. 2008; Jakob et al. 2009, 2010) to detect geographic patterns that might be caused by Holocene climate changes. Our study investigates 1) whether a regional substructure exists in the extant wild barley genepool, that is, how genetic diversity is distributed throughout its natural distribution area; 2) processes underlying the present geographic and genetic distribution patterns of wild barley, based on phylogeographic data and ecological predictive models; and 3) the influence of ex situ conservation on genetic structure in wild barley accessions.

## Materials and Methods

### Plant Materials

In total, 415 genotypes were studied (supplementary table S1, Supplementary Material online). We included 123 individuals of wild barley, *H. vulgare* L. subsp. *spontaneum* (C. Koch.) Thell., which were freshly collected from 78 collection sites in ten countries between 1999 and 2011 (“wild *H. spontaneum*”). None of these accessions was collected at a road site or near cultivated barley fields. Twenty-three individuals were collected in the west of the distribution area (Israel, Palestine, Jordan, Syria, and Greece); among them, 19 were chosen from the B1K collection (Hübner et al. 2009). Eighty individuals (42 collection sites) were selected from the natural distribution range in Turkey. Twenty individuals were included from the eastern part of the range (Iran, Tajikistan, and Uzbekistan).

For ex situ-propagated materials, we studied 152 subsp. *spontaneum* individuals (“genebank *H. spontaneum*”) belonging to 62 distinct accessions. This material was mainly collected between the 1950s and 1990s and has undergone multiple propagation cycles ex situ. Thirty-five accessions of genebank *H. spontaneum* were selected from the United States Department of Agriculture, Agricultural Research Service (USDA-ARS), among them the 25 subsp. *spontaneum* accessions were investigated by Lin et al. (2001), Morrell et al. (2003), and Morrell and Clegg (2007). We included seeds from two different seed orders of identical accession numbers from the same genebank, and accessions that were exchanged among genebanks. In the latter cases, we ordered accessions having the same identifier from USDA-ARS and the International Center for Agricultural Research in Dry Areas (ICARDA).

Finally, we included 137 lines of domesticated barley, *H. vulgare* L. subsp. *vulgare*, consisting of 82 barley cultivars from 31 countries and 55 barley landraces from 30 countries. Furthermore, three six-rowed accessions (f. *agriochrithon* [Åberg] Bowd.) were considered.

### DNA Amplification, Sequencing, and Sequence Analysis

Seven single-copy loci were sequenced to infer the evolutionary history of wild barley. Genomic DNA extraction, PCR amplification, purification, and sequencing of six out of the seven loci (*ADH2*, *ADH3*, *AMY1*, *DHN9*, *GAPDH*, and *PEPC*) followed the protocols given by Kilian et al. (2006). For the *PPD-H1* locus on chromosome 2HS (Turner et al. 2005) and for further details, see supplementary text S1 and tables S2 and S3, Supplementary Material online.

### Network Reconstruction and Genetic Clustering

Median-Joining (MJ) networks (Bandelt et al. 1999) for haplotypes at each locus were constructed with the software programs DNA Alignment 1.3.1.1, Network 4.6.1.0, and Network publisher 1.3.0.0 (Fluxus Technology Ltd., Clare, Suffolk, UK). 

We performed Bayesian cluster assignment analyses to infer the spatial structure in the genetic data of 123 wild *H. spontaneum* individuals using Structure 2.3.3 (Pritchard et al. 2000) and utilized a second approach that incorporates spatial information, as implemented in Geneland version 3.2.4 (Guillot et al. 2005) for R 2.11.1 (R Development Core Team 2011) (supplementary text S1, supplementary table S4, Supplementary Material online).

### Genetic Differentiation and Genetic Diversity among and within Inferred Clusters

For inferred Structure and Geneland clusters, pairwise population estimates of genetic differentiation were calculated from allele frequencies using Jost’s *D*, which is independent of heterozygosity (Jost 2008), estimated as the harmonic mean of the pairwise mean values for each of the seven loci using Smogd (Crawford 2010). For comparison, Weir and Cockerham’s (1984) *F*_ST_ was calculated using Fstat 2.9.3 (Goudet 1995) and pairwise 95% confidence intervals for F_ST_ were estimated from 15,000 bootstrap resamplings (supplementary tables S5 and S6, Supplementary Material online). To allow comparisons of genetic diversity at all loci among the inferred clusters within wild *H. spontaneum*, genebank *H. spontaneum*, and domesticated barley, allelic richness *R* was estimated considering rarefaction (Gotelli and Colwell 2001) for a corrected sample size of *n* = 10 (Structure) and *n* = 5 (Geneland), due to distinct group sizes, using a Matlab script (Jakob et al. 2007). Nucleotide diversity *H* (Nei 1987) was calculated by applying Fstat 2.9.3 (Goudet 1995) (supplementary tables S7 and S8, Supplementary Material online).

### Species Distribution Models: Potential Extant and Paleodistributions 6,000 and 21,000 Years Ago

We compiled a set of occurrence data from 360 point localities for subsp. *spontaneum*, using data from different data sources (see supplementary text S1, Supplementary Material online, for details). A subset of bioclimatic variables obtained from www.worldclim.org (last accessed March 20, 2014) (vers. 1.4, Hijmans et al. 2005), checked for low multicolinearity and assumed to be relevant in subsp*. spontaneum*, was extracted for present climatic conditions, for the last glacial maximum (LGM) 21,000 years before present (yBP), and for the mid-Holocene climate optimum (MH) 6,000 yBP, each for two different global circulation models (see supplementary text S1, Supplementary Material online). In brief, for species distribution model (SDM) computation, we used an ensemble model approach based on eight single model algorithms as implemented in Biomod for *R* (Thuiller 2003; Thuiller et al. 2009). Detailed descriptions of SDM settings can be found in the supplementary text S1, Supplementary Material online.

## Results

### Genetic Diversity at Seven Nuclear Loci

**Fig. 1.—:**
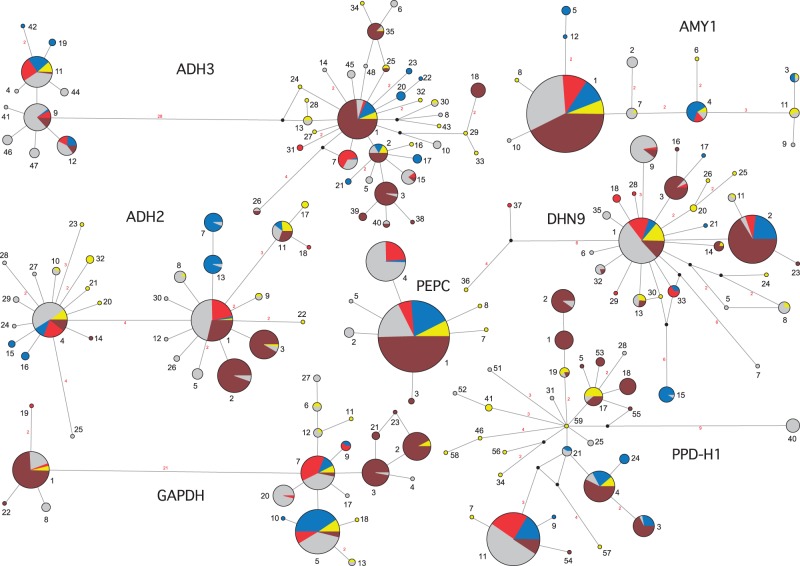
MJ networks derived from resequenced DNA haplotypes at seven loci of all 415 lines studied. Circle sizes correspond to the frequency of that particular haplotype. Yellow, wild *H. spontaneum* accessions assigned to the Western Cluster; blue, Turkish Cluster; red, Eastern Cluster; gray, genebank *H. spontaneum* accessions; and brown, domesticated barley incl. f. *agriocrithon*. Median vectors are indicated by black dots. Haplotype numbers are given (black numbers). Distances between haplotypes are indicated in basepairs (red numbers; only distances >1 bp are shown). Alignment gaps were not considered. Heterozygotes were not considered.

All loci were polymorphic in subsp. *spontaneum*, and six out of seven were polymorphic in domesticated barley. Among the 123 wild *H. spontaneum* accessions, we found between 4 (*PEPC*) and 28 (*ADH3*) distinct haplotypes per locus (fig. 1 and table 1). Generally, allelic richness *R* (for *n* = 10) was highest at *ADH3* (6.56) and lowest at *PEPC* (2.38). Two loci (*AMY1*, *PEPC*) showed low allelic richness (nine and four haplotypes, respectively; *R* < 3). Three loci (*DHN9*, *GAPDH*, *PPD-H1*) revealed medium-level allelic richness (*R* values between 3 and 5), and two loci (*ADH2*, *ADH3*) possessed high *R* values (18 and 28 haplotypes, respectively; *R* > 5) (table 1).

**Table 1:** Genetic Diversity Recorded at Seven Barley Loci Based on Structure Analysis (*K* = 3)

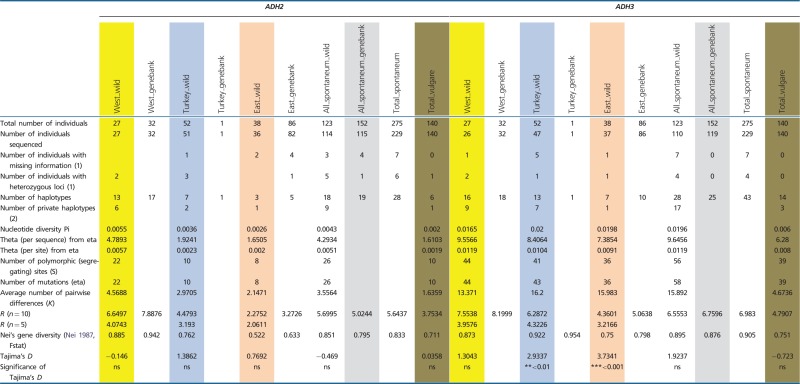
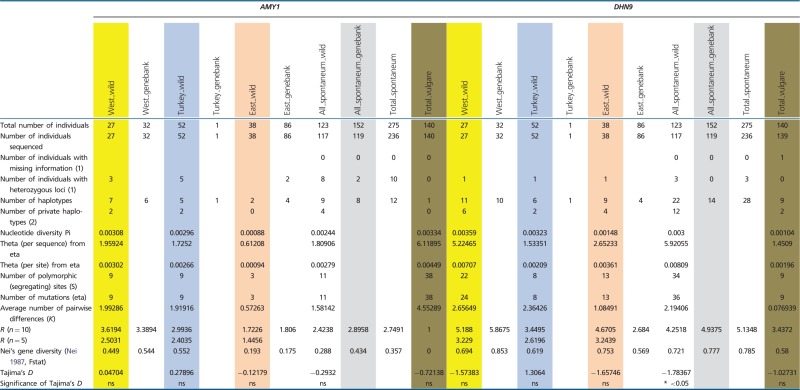
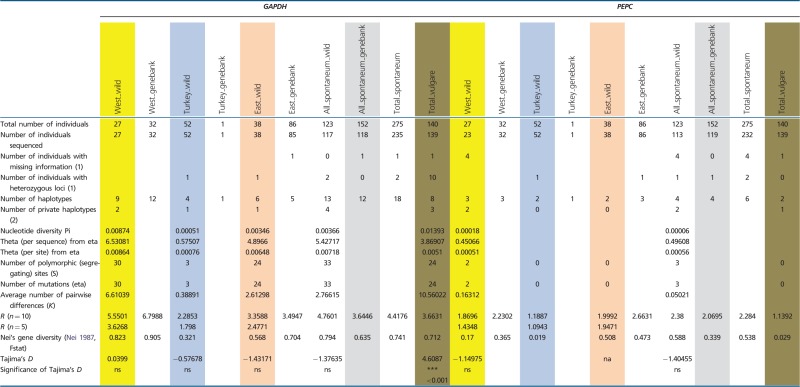
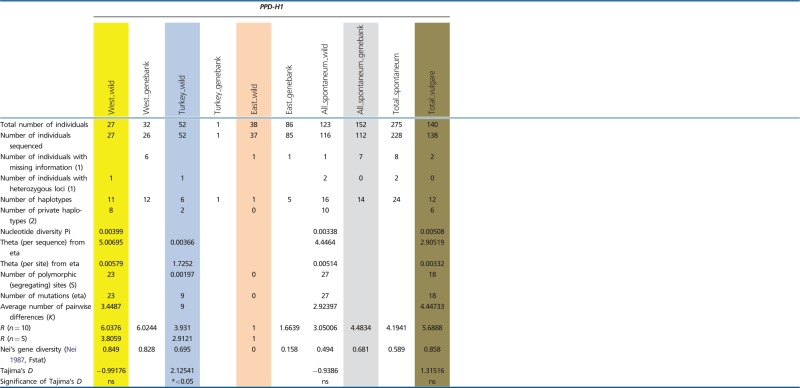

Note.—Data are shown for three groups of wild *H. spontaneum*, genebank *H. spontaneum* and domesticated barley accessions. West_wild, Western Cluster; Turkey_wild, Turkish Cluster; East_wild, Eastern Cluster; West_genebank, genebank accessions assigned to the wild Western Cluster; Turkey_genebank, genebank accessions assigned to the wild Western Cluster; East_genebank, genebank accessions assigned to the wild Western Cluster; Ns, not significant. (1) Admixed individuals not considered. (2) genebank *H. spontaneum* accessions not considered.

Similar patterns of haplotype numbers per locus were found in the 152 genebank *H. spontaneum* samples. Between 4 (*PEPC*) and 25 (*ADH3*) distinct haplotypes per locus were detected. Haplotype richness *R* (for *n* = 10) was highest at *ADH3* (6.76) and lowest for *PEPC* (2.07). At most loci, the *R*-value and Nei’s gene diversity *H* were higher than in wild *H. spontaneum* samples (table 1). In both wild and genebank *H. spontaneum*, only very few haplotypes per locus (mostly one to two) reached higher frequencies (>20% of the analyzed samples; supplementary table S3, Supplementary Material online). Up to 90% of the haplotypes per locus were rare, showing frequencies below 10% and even below 5%. Thus, in subsp. *spontaneum*, a general pattern of few frequent haplotypes per locus along with a series of many alleles at low frequencies was found (fig. 1).

The analyzed domesticated barley individuals possessed fewer alleles than their wild progenitor at all loci. Between one haplotype at *AMY1* and 14 haplotypes at *ADH3* were found. Accordingly, allelic richness *R* and gene diversity *H* were generally lower in domesticated barley than in subsp. *spontaneum*, with the exception of *PPD-H1,* where both values were higher in domesticated barley. Some haplotypes in domesticated barley reached frequencies between 40% and 100%. However, these major haplotypes are not necessarily the most frequent alleles in its wild progenitor. Major barley alleles are largely absent in wild *H. spontaneum* (e.g., HT02 at *ADH2*, HT03 at *ADH3*, HT03 at *GAPDH*, HT01 at *PPD-H1*) or very rare (e.g., HT03 at *ADH2*, HT03 at *DHN9*) (supplementary table S3, Supplementary Material online). At each locus, between 1 (*PEPC*) and 6 (*PPD-H1*) private alleles (except at *AMY1*, where HT1 is fixed) were observed, some at high frequencies. These private barley alleles were not detected in the genepool of its wild progenitor (fig. 1; supplementary table S3, Supplementary Material online).

### Population Structure within Wild *H. spontaneum*

In order to reveal the underlying population structure of wild *H. spontaneum,* three analyses approaches were used.

#### Structure Analysis

**Fig. 2.—:**
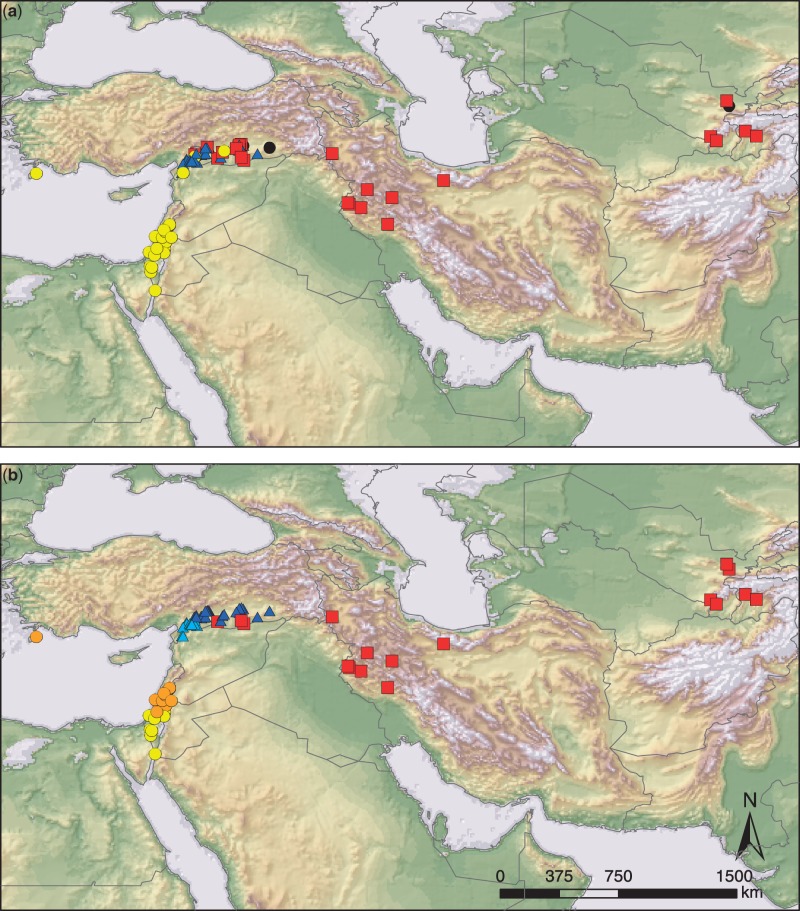
Geographic information system (GIS)-based topographic maps indicate the natural distribution of 123 genotypes of wild *H. spontaneum* based on (*a*) Structure (*K* = 3)-inferred clusters. Yellow circles, wild *H. spontaneum* accessions assigned to the Western Cluster; Blue triangles, Turkish Cluster; Red squares, Eastern Cluster; black circles, hybrids; (*b*) Based on Geneland (*K* = 5)-inferred clusters. Yellow circles, “Southern Levant Cluster”; orange circles, “Northern Levant Cluster”; light blue triangles, “Southern Turkish Cluster”; dark blue triangles, “Southeastern Turkish Cluster”; red squares, Eastern Cluster.

For the initial Bayesian assignment analyses of 123 wild *H. spontaneum* individuals using Structure with *K* varying from 1 to 10 and Structure Harvester, we found for Δ*K* (Evanno et al. 2005) a strong peak at *K* = 3. After repeating the clustering procedure ten times with *K* fixed at three, individuals were assigned to their most likely genetic group using Clumpp at a threshold of 0.6. Most individuals (95%) were assigned unambiguously to one of the three clusters (supplementary fig. S1 and table S4, Supplementary Material online). These genetic clusters were geographically clearly separated (fig. 2). One cluster (*n* = 27) encompassed individuals collected in the western part of the distribution area, that is, Greece, Israel, Lebanon, Jordan, Syria, and southern Turkey (“Western Cluster”). Another (*n* = 52) consisted of individuals collected exclusively in southeast Turkey (“Turkish Cluster”). A third (*n* = 38) contained individuals collected in the eastern part of the distribution range and reached from Turkey throughout Iran into Uzbekistan and Tajikistan (“Eastern Cluster”). All three clusters cooccurred and overlapped in Turkey. Six admixed individuals were detected. Among these, five nonassigned individuals originated in Turkey and were considered hybrids between two clusters (supplementary table S4, Supplementary Material online). No clear geographic outliers, that is, individuals with allele compositions not belonging to their geographic region of origin, were detected within these three genetically defined clusters for wild *H. spontaneum* individuals. Mostly, only the major haplotypes at each locus were shared among the three clusters. Genealogical networks showed that these shared alleles are located at central interior positions, indicating that they are ancient alleles (fig. 1). The amount of private haplotypes at each locus, that is, alleles restricted to one of these three clusters, was high, and ranged between 30% at *PEPC* and 61% at *PPD-H1* (table 1).

#### Geneland analysis

The posterior density and log-likelihood levels of all chains stabilized long before the end of the run and showed that 3 million sampling generations were sufficient to reach convergence. In the initial run, 23 out of 25 chains converged at *K* = 6 (92% of all chains). The remaining two iterations converged at *K* = 5. Thus, *K* was fixed at 6 for the subsequent runs. However, one cluster was not assigned to any of the individuals and therefore formed an artifact (empty cluster) with a low distribution of posterior probability, superimposed by the remaining clusters. Although minor differences were apparent in the assignment of single individuals, the results obtained by Geneland were congruent with the results obtained by Structure (supplementary table S4, Supplementary Material online). Clearly defined clusters of wild individuals were observed (fig. 2). The Eastern Cluster (*n* = 26) conformed that of the Structure analysis and covered a huge geographic area, reaching from the southern part of the Turkish province Urfa throughout Iran into Uzbekistan and Tajikistan. The Structure-derived Turkish Cluster was further subdivided by Geneland. The first cluster occurred in the provinces Hatay and Gaziantep, providing the connection to accessions from the western part of the Fertile Crescent (further named southern Turkish cluster = “S Turkish Cluster,” *n* = 22). The second cluster consisted of accessions sampled further east throughout the provinces Gaziantep, Adiyaman, Urfa, Diyarbakir, and Mardin (southeastern Turkish cluster = “SE Turkish Cluster,” *n* = 52). The Structure-derived Western Cluster was divided into one group of individuals collected in southern Israel and Jordan (southern Levant cluster = “S Levant Cluster,” *n* = 9) and a second group occurring further north in Israel, Syria, Jordan, Lebanon, and Rhodes/Greece (northern Levant cluster = “N Levant Cluster,” *n* = 14) (fig. 2).

#### Neighbor-Net Analysis

Congruent evidence for the population structure in wild *H. spontaneum* genotypes was provided by NeighborNet planar graphs and neighbor-joining trees (supplementary figs. S2 and S3, Supplementary Material online). All five clusters obtained by Geneland analysis were distinguishable, although a high amount of reticulation was evident, owing to the fact that common alleles per locus were shared among geographic regions. Both analyses showed a certain overlap of the Turkish genotypes with either the Western Cluster or the Eastern Cluster.

### Genetic Diversity and Genetic Differentiation within and among Inferred Clusters of Wild *H. spontaneum*

Based on the allelic richness and Nei’s gene diversity values for wild *H. spontaneum*, all seven nuclear loci showed a general trend of decreasing genetic diversity from the western part of the species’ area eastward. For *Adh2*, *Adh3*, *AMY1,* and *PPD-H1,* both values were lowest in the eastern part. At *Dhn9*, *GAPDH,* and *PEPC* both estimates were lowest in Turkey. Private haplotypes, that is, haplotypes restricted to a certain region, occurred in all three geographical clusters. However, the majority of them were found in the Western Cluster (table 1), mostly at low frequencies. Unique alleles were also found in the Turkish Cluster, although at reduced numbers per locus (0–57%), and were rare in the Eastern Cluster (0–44%) (supplementary table S4, Supplementary Material online).

Levels of genetic differentiation among the three geographic regions inferred by Structure were significant (*D* and *F*_ST_) and the following trend was visible: differentiation was largest between the “Turkish” and the “Eastern” clusters (0.31; 0.26**), and lowest between the Turkish and the “Western” clusters (0.22; 0.15**) (supplementary table S5, Supplementary Material online). In the finer resolved clustering of Geneland, the pattern was more complex and should be noted with caution because of the limited sample size per cluster (supplementary table S6, Supplementary Material online). In general, geographically adjacent clusters were less differentiated than geographically more distant groups.

### Assigning Domesticated Barley Accessions to Three Predefined Wild *H. spontaneum* Clusters

To understand the interrelationship between barley and its wild progenitor, that is, if subsp. *spontaneum* from specific geographic regions contributed particularly to the genetic composition of the domesticated crop, we assigned barley accessions to the predefined wild *H. spontaneum* clusters obtained by Bayesian assignment analyses. The clustering results of Geneland and Structure for wild *H. spontaneum* were congruent. However, due to the higher number of Geneland clusters, some groups contained only few individuals. Therefore, we assigned domesticated barley individuals only to the three Structure-inferred clusters.

Based on the seven analyzed loci, most of the 137 domesticated barley individuals revealed rather mixed ancestry. Only ∼51% of barley individuals were assigned unambiguously to any of the three clusters applying the 60% ancestry criterion (supplementary fig. S1 and table S4, Supplementary Material online). However, there is no geographic association evident with this assignment. Sixty-nine individuals from 36 countries were assigned to the Western Cluster. Three were included in the Eastern Cluster (from Japan, South Korea, and Syria). No domesticated barley line was assigned unambiguously to the Turkish Cluster. In general, the contribution of the Turkish Cluster (mean contribution, 24 ± 11%) and the Eastern Cluster (mean contribution, 18 ± 10%) to the genepool of the diverse set of domesticated barleys investigated was rather low compared with the contribution of the Western Cluster (mean contribution 58 ± 11%). However, barleys from Middle East (Iran, Iraq, Kyrgyzstan, and Turkmenistan) and landraces from Turkey in particular showed a relatively higher relation with Turkish wild *H. spontaneum* lines (mean contribution, 38 ± 5%) compared with the remaining barley lines (mean contribution, 20 ± 7%).

### Assigning Genebank *H. spontaneum* to Predefined Wild *H. spontaneum* Clusters

To obtain an overview about the geographic representation and population structure of *H. spontaneum* stored in genebank repositories, we included 152 genebank *H. spontaneum* individuals. Several individuals per genebank accession number were considered and assigned to the predefined clusters of wild *H. spontaneum* inferred by Structure. To a large extent, the genebank *H. spontaneum* samples fitted into the predefined clusters, strengthening the geographic pattern of wild barley cluster distribution (supplementary fig. S4, Supplementary Material online). However, 33 individuals (22%) could not be assigned unambiguously to any of the three Structure clusters applying the 60% ancestry criterion, indicating admixed ancestry. Changing the MIGPROR setting of Structure did not change this high proportion of admixed individuals (results not shown). In contrast to wild *H. spontaneum*, where such hybrids were rare (<5%) and restricted to Turkey where the three clusters overlap, the 33 admixed genebank *H. spontaneum* individuals were found among 20 genebank accessions. These accessions originated, based on their passport data, mainly from Iraq, Iran, and Afghanistan, but also from Libya, Syria, Egypt, Jordan, and Turkey, and were identified mainly as hybrids between the Western and Eastern Cluster (supplementary table S4, Supplementary Material online).

While analyzing wild *H. spontaneum* samples, we did not encounter any geographic outlier, whereas 12 individuals of genebank *H. spontaneum* samples were not admixed but geographically misclassified (supplementary table S4, Supplementary Material online). In most cases, according to their passport data, these samples originated from Iran but were genetically unambiguously clustered into the Western Cluster (supplementary fig. S4, Supplementary Material online).

As multiple individuals were sequenced for most genebank *H. spontaneum* accessions, we were able to study intra-accession genetic diversity. It became obvious that several genebank accessions consisted of a mixture of different genotypes, which were partly assigned to different genetic/geographical clusters. In addition, several genotypes from the same accession number were identified as admixed without assignment to any cluster, whereas others were assigned unambiguously to one of the three clusters. In some cases, the genotypes within one accession differed at up to six out of seven loci analyzed (supplementary table S2, Supplementary Material online). Several accessions studied by Morrell et al. (2003) belonged to genetically/geographically misclassified or admixed materials (e.g., PI 219796, PI 254894). Furthermore, for the same accessions, we observed considerable genetic differences between detected genotypes from newly ordered seeds (ordered by S.S.J. in 2011) and 1) sequences released by Morrell et al. (2003) or 2) individuals from previous seed orders (e.g., before 2000, as part of the Badr et al. [2000] collection, or before 2008 ordered by B.K.) (supplementary table S1, Supplementary Material online).

### Current Distribution and Inference of Distribution 6,000 and 21,000 Years Ago

**Fig. 3.—:**
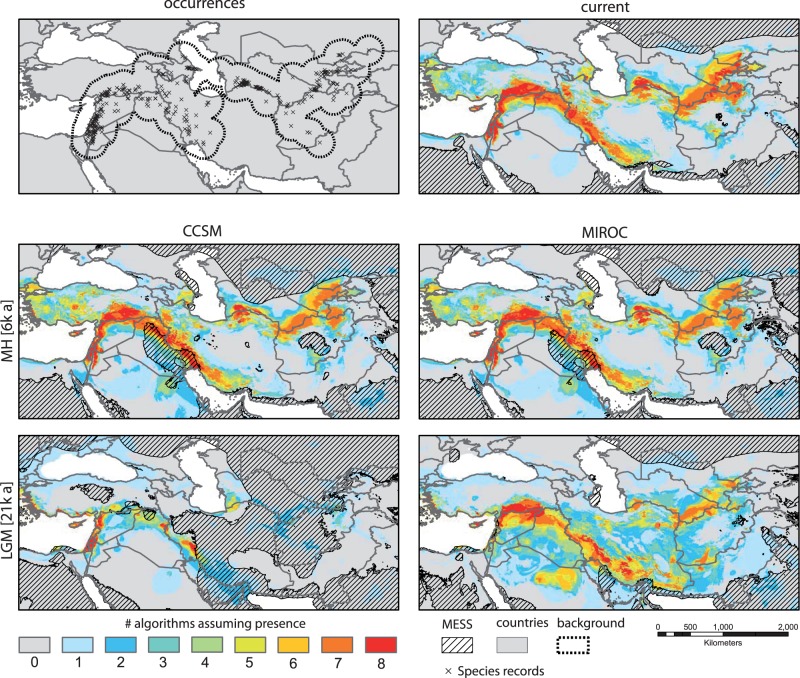
Potential current and paleodistributions of wild barley. Three hundred sixty point localities for subsp. *spontaneum* (upper left) and current distributional predictions (upper right) are shown. Distributions of suitable climatic conditions for subsp. *spontaneum* during the mid-Holocene climate optimum (MH) about 6,000 yBP (6ka) and for the last glacial maximum (LGM) about 21,000 yBP (21ka) using CCSM and MIROC climate models are presented. Areas indicated as MESS comprise nonanalogous climate conditions with reference to the training range of the SDM.

The potential distributions derived from the SDM ensemble are given in figure 3. As expected when applying multiple algorithms, model performance varied among the eight techniques (Elith et al. 2006). The predictive performance of the SDMs was “excellent” in terms of area under the curve (AUC; mean_AUC_ = 0.92) and True Skills Statistic (TSS; mean_TSS_ = 0.72) and “moderate” in Kappa statistics (mean_Kappa_ = 0.52) (supplementary table S9, Supplementary Material online), leading to different weights of single algorithms in the final ensemble model (supplementary table S10, Supplementary Material online).

Relative contributions of environmental predictors varied across different algorithms (supplementary table S11 and fig. S5, Supplementary Material online). In general, variables describing temperature and precipitation regimes during extreme quarters (i.e., driest, coldest, and wettest quarter) appeared to restrict the potential distribution. Here, precipitation during the coldest quarter (BIO19) was by far the most important variable, followed by precipitation during the wettest (BIO16) and driest quarters (BIO17), as well as mean temperature during the driest (BIO9) and coldest quarter (BIO11). All other variables used here (i.e., BIO 2, 7, 8, 10, and 18) had an overall low importance across all algorithms (not shown; for abbreviations see supplementary table S9, Supplementary Material online).

The current distributional predictions (fig. 3) were fairly good representations of the taxon’s extant geographical distribution (Harlan and Zohary 1966; Bothmer et al. 1995). This is also represented by the relatively high contribution of environmental predictors describing temperature and precipitation regimes during extreme quarters. Thus, suitable climatic conditions for wild barley were predicted at high probabilities in the Mediterranean climate of the Fertile Crescent. East of the Zagros Mountains (dry Iranian plateau) the species was only sparsely predicted along the northerly adjunct mountainous area west of the Caspian Sea. The second main distribution area with suitable climate conditions in the East encompasses semiarid areas with humid and mild winters southeast of the Caspian Sea in the Kobet dag Mountain area between Iran and Turkmenistan, along the mountainous areas of northern Afghanistan, southern Turkmenistan, and Uzbekistan toward the Pamir region of Tajikistan. Additionally, the models predicted the species on Cyprus and along the Mediterranean coast of Turkey. As expected, subsp. *spontaneum* clearly avoids the rather dry and hot, partly desertlike areas of Central Syria and Iraq, the southern plains of Euphrates and Tigris, and Central Iran, as also indicated by the relative contributions of the environmental predictors.

The distribution of suitable climatic conditions for subsp. *spontaneum* during the Holocene climate optimum in the Atlanticum about 6,000 yBP coincided highly in both climate models (CCSM and MIROC, supplementary text S1, Supplementary Material online) with the current situation (fig. 3). Only slight differences were observed in Central Turkey and the eastern part of the distribution area.

Maps showing the potential paleodistribution of subsp. *spontaneum* for the LGM about 21,000 yBP are given for both Pleistocene climate models used (fig. 3). However, comparing both models in terms of predictions of potential species distribution during the LGM revealed strong differences. MIROC predicted rather large potential Pleistocene distribution along the southern foothills of the Taurus Mountains and southwestern foothills of the Zagros Mountains. Although predominantly nested within the MIROC projections, CCSM on the other hand predicted wild barley during the LGM nearly exclusively in the eastern Mediterranean, in the Turkish Aegean region, and on Cyprus. Two additional small areas, where the species might have occurred with high probability 21.000 yBP, were predicted from both models: 1) around the provinces As-Sulaimaniyya/Diyala (Iraq) to Kermanshah (Iran) and 2) in the Iranian province Khuzestan.

## Discussion

We present the first phylogeographic analysis of *H. vulgare* subsp. *spontaneum* throughout its distribution area in Southwest and Middle Asia, based mainly on materials freshly collected during the last decade from wild stands. In contrast to earlier studies (Morrell and Clegg 2007; Russell et al. 2014), we used genebank accessions only to fill sampling gaps and to compare allele compositions. With our phylogeographic analysis at seven resequenced nuclear loci in wild barley, we arrived at six key findings.

### The Genepool of Wild *H. spontaneum* Consists of Three Main Clusters

We inferred the population structure of wild *H. spontaneum* by Bayesian assignment analysis using Structure and Geneland (thus integrating geographic information as well) and computing phylogenetic networks. These analyses provided congruent evidence for the occurrence of three major clusters in the wild barley genepool. A Western Cluster occurs throughout the Levant and southern Turkey; a Turkish Cluster comprises large parts of southeastern Turkey, and an Eastern Cluster reaches from Turkey into Middle Asia. This is in contrast to findings by Morrell and Clegg (2007), who identified only a western and an eastern genepool, probably due to the lack of Turkish populations in their analysis. Russell et al. (2014), using 256 wild barleys maintained at ICARDA and largely lacking Turkish materials, identified five clusters using biallelic SNPs. In the Geneland analysis, our Western Cluster was further subdivided. This bipartition is largely in accordance with results of Hübner et al. (2012) who, by analyzing 215 individuals of subsp*. spontaneum* from Israel with microsatellite and biallelic SNP markers, found three genepools, differentiating wild barleys from three ecogeographic regions (northern Levant, Mediterranean coast, and desert). Our N Levant Cluster roughly coincides with their northern and coastal types whereas the S Levant Cluster represents their desert type.

In Turkey, the three major clusters inferred by Structure overlap geographically (fig. 2). Using Geneland and NeighborNet analysis, two subclusters of the Turkish Cluster were found. Higher mountain ranges or other current topographic barriers do not separate the distribution areas of these subclusters. Therefore, we assume that they either reflect two ecotypes, which evolved along increasing continental climate conditions toward the east or resulted from different refugial populations. Limited gene flow and different effective population sizes could be the reason for their current separation (Hübner et al. 2012). The region adjacent to the northeastern end of the Mediterranean Sea that is inhabited by the S Turkish Cluster was among the areas predicted at high probabilities as potential refugia during the LGM. Support for the peculiarity of the region southwest of Gaziantep comes also from wild einkorn (Kilian et al. 2007) and wild emmer wheat (Özkan et al. 2011), where the same region was identified as harboring unique genepools.

### Wild *H. spontaneum* Samples Show Strong Correlation between Genotype and Geographic Origin

All wild *H. spontaneum* individuals were assigned to one of the three clusters by Structure according to their geographic region, and only 5% of the individuals showed genotypes indicative for admixed ancestry. These “hybrid” individuals originated from Turkey, where the Turkish Cluster overlaps with the other two Structure-based clusters. Regarding haplotype distribution, the three major clusters are, however, not completely separated but share particularly the oldest haplotypes at all analyzed loci. Younger haplotypes, mostly occurring at lower frequencies, were unique to geographical areas. This is a typical pattern of incomplete lineage sorting (Jakob and Blattner 2006), that is, the origins of these oldest haplotypes predate the separation of the extant geographically separated groups, while younger alleles originated within these groups and are still restricted to their original areas. In case of recent gene flow between groups, we would also expect younger haplotypes to be shared among regions.

As already observed by Lin et al. (2001) at *Adh3* and by Morrell et al. (2003), at three out of the seven loci studied here (*ADH2*, *ADH3*, and *GAPDH*), two major groups of haplotypes were observed, with 28 inferred mutational steps separating both haplotype groups at *ADH3*, 21 at *GAPDH*, and four at *ADH2*. Even our much larger sample in comparison to Morrell et al. (2003) did not reveal intermediate types. These haplotype groups were not associated with a geographical cluster and both types occur also in domesticated barley. Cloning and sequencing of amplicons as well as syntheny-based searches in sequenced grass genomes (Mayer et al. 2011; International Barley Genome Sequencing Consortium 2012) of these loci gave no indication of paralogs. Genetic differences between these groups of haplotypes exceed by far the genetic distances found within these groups. This can be explained either by an old split of the subsp. *spontaneum* genepool, predating the origin of the three groups inferred in this study and merging of the groups after secondary contact, or by postulating that one of the haplotype groups was obtained by introgression of a distantly related taxon (Mahelka and Kopecky 2010; Brassac et al. 2012) early in the species’ history. Surprisingly, we found only few recombinant haplotypes at these loci, indicating suppressed recombination in their respective genomic areas, which might be the key feature for the prolonged maintenance of the different sequence types.

### The Highest Genetic Diversity in Wild *H. spontaneum* Occurs in the Levant

The Western Cluster possesses the highest genetic diversity and numbers of private haplotypes at all loci (*n* = 35), although these alleles occur mostly at low frequencies. Although a moderate number of private haplotypes (*n* = 16) is present in Turkey, the number of private haplotypes generally decreases toward the east (*n* = 7). This pattern of distribution of genetic diversity is compatible with the long-term occurrence of wild barley in the western parts of its distribution area and a relatively more recent occurrence in areas east of Turkey. However, the pattern of haplotype diversity in the Western Cluster of wild *H. spontaneum* is unusual, as old and widespread alleles do not dominate in this area and singletons were found frequently. Although our sample size of wild *H. spontaneum* in the western part of its distribution area is relatively low, the lack of old and dominating haplotypes is unlikely to be a sampling artifact, as our sampling was conducted randomly. Thus, we predict that further increasing the sample size of wild *H. spontaneum* in the Levant will detect more individuals carrying major haplotypes and also more haplotypes occurring at low frequencies. Such a haplotype pattern can be explained by an overall large effective (meta) population size in the area together with predominant inbreeding in highly structured populations. The high topological diversity of the western Levant, with several mountain ridges separated by lowlands with Mediterranean to arid climate, seems to have been predestined to function as a biodiversity pump during Pleistocene climate cycles, when populations split and reunified repeatedly. In contrast, in more northerly or continental habitats, the Pleistocene glacial maxima resulted in repeated severe bottlenecks for plant populations, reducing genetic diversity or even causing their extinction. This difference must be considered, when we discuss refugia in Southwestern Asia, that is, that refugia at this southerly latitude were much larger in comparison to northern habitats, and that their impact on the species’ genepool was mostly due to substructuring of genetic variation instead of reduction to a few surviving genotypes.

### Genebank Materials of subsp. *spontaneum* Seem Often to Be Introgressed

Genebank *H. spontaneum* showed a significantly higher degree of admixed geographical ancestry (22%) in comparison with wild *H. spontaneum* individuals (5%). Origins of such admixed accessions were not restricted to Turkey, where clusters overlap, but originated from all over the distribution area. Moreover, clustering of genebank *H. spontaneum* accessions resulted in several cases in conflict between the reported area of origin and their assignment to geographic clusters. As these phenomena were nearly absent in recently collected wild materials, hybridization has most probably taken place during propagation and maintenance in ex situ seed repositories. Intra-accession diversity, found through sequencing of multiple individuals per genebank accession, was observed. This might also indicate a certain amount of admixture due to ex situ germplasm handling.

The classification of subsp. *spontaneum* as strong inbreeder (Abdel-Ghani et al. 2004) obviously resulted in handling protocols for genebanks (i.e., separation of different accessions by only few meters distance during multiplication, wild and domesticated cereals next to each other) that do not prevent hybridization. Moreover, we cannot discern if the wrongly clustered accessions behaved in this way due to hybridization or if some additional mixup of seeds or passport data had taken place. The only other explanation for the observed patterns would be a substantial change in genetic diversity in natural populations during the last few decades, that is, that materials collected half a century ago were genetically more diverse than today and that most of this diversity was lost in natural populations during the last decade but maintained in genebanks. Although such an argument might explain the high intra-accession diversity of old genebank materials, it does not account for the clear geographic structure found in recently collected populations of wild *H. spontaneum*, which is absent in genebank *H. spontaneum*. It is hardly conceivable that lineage sorting could be so rapid, mutually extinguishing “wrong” alleles from all three major geographic clusters. Therefore, the likely explanation is hybridization among genebank accessions during rounds of ex situ multiplication and/or mixup of seeds or passport data during repeated seed exchange among different genebanks.

These findings imply that subsp. *spontaneum* accessions obtained from genebanks, at least those that have been through several propagation cycles or exchanged between genebanks, are mostly not suitable to study phylogeographic patterns. Therefore, recently collected materials from naturally occurring populations are required. To maintain such lineages ex situ*,* reasonable diligence is necessary to prevent cross-pollination, which means that cereal species assumed to be inbreeding should be treated in genebanks similar to outbreeding species.

### The Haplotype Composition of Wild *H. spontaneum* and Domesticated Barley Was Found to Be Different

The diverse set of 140 domesticated barleys of worldwide origin (including f. *agriocrithon*) possessed fewer haplotypes (52 haplotypes) at all loci than wild *H. spontaneum* (110 haplotypes). Thus, nucleotide diversity in domesticated varieties is lower than in the wild progenitor, a result that is in accord with a proposed domestication bottleneck (Caldwell et al. 2006; Kilian et al. 2006). However, haplotype frequencies in wild and domesticated lineages were quite different, that is, cultivated barley possesses haplotypes at most loci that were rare or absent in wild *H. spontaneum*. One explanation could be that adaptation of the crop to new environments outside the natural distribution range of the species resulted in the selection of favorable alleles, which are not present in subsp. *spontaneum* or only at very low frequencies. Evidence for selection of preexisting haplotypes from the wild barley genepool and subsequent enrichment in domesticated barley was found for HT01 at *ADH2*, HT01 at *ADH3* (most probably selected from the Western or Turkish Cluster), HT01 at *AMY1*, HT02 and HT03 at *DHN9,* and HT04 at *PPD-H1*. Another explanation is that a wild genepool different from extant subsp. *spontaneum* was domesticated and completely absorbed by the domesticated form. In this case, allelic differences between barley and wild *H. spontaneum* would reflect an older pattern that can no longer be detected in the wild.

In contrast to wild *H. spontaneum*, genebank *H. spontaneum* often shared haplotypes with domesticated barley accessions (e.g., HT02 at *ADH2*, HT03 at *ADH3*, HT03 at *GAPDH*, and HT02 at *PPD-H1*). Although hybridization between subsp. *spontaneum* populations and barley was reported for the Levant where they occur in close proximity (Hübner et al. 2012), the rarity of barley alleles in our sample of wild *H. spontaneum* again indicates that this pattern might have originated through introgression during ex situ-propagation.

These results have far-reaching consequences. If gene flow between subsp. *spontaneum* and domesticated barley occurs in situ (Hübner et al. 2012) or ex situ during seed multiplication in genebanks, as shown here, inclusion of such accessions in evolutionary or domestication studies can result in wrong outcomes regarding the region and mode of domestication of the crop. Because introgressed accessions have higher similarity to the crop than true wild *H. spontaneum*, previous studies, particularly in the field of barley domestication (e.g., Badr et al. 2000; Kilian et al. 2006; Morrell and Clegg 2007; Saisho and Purugganan 2007; Jones et al. 2008), should be considered with caution, as they were based exclusively or to a large extent on materials obtained from genebanks.

### Refugia of Wild Barley About 21,000 Years Ago Were within the Eastern Mediterranean, Particularly in the Levant

Projecting the SDM onto palaeoclimatic reconstructions suggested by CCSM and MIROC, the model suggests only marginal differences between the species’ current potential distribution and those projected for 6,000 yBP. However, for 21,000 yBP, the potential distributions show pronounced differences that are much larger according to MIROC than to CCSM. These differences in the size of potential distributions are conceptually consistent with projections developed for other European and western Asian taxa (Garcia-Porta et al. 2012; Rebelo et al. 2012; Schorr et al. 2012; Tarkhnishvili et al. 2012) and can be traced back to differences in the paleoclimatic conditions suggested by CCSM and MIROC. Although CCSM suggests a trend in surface air temperature of −0.012 °C/century under current conditions, −0.007 °C/century at 6,000 yBP, and −0.010 °C/century at 21,000 yBP, MIROC suggests 6e−04 °C/century under current conditions, −0.022 °C/century at 6,000 yBP, and −0.050 °C/century at 21,000 yBP. These differences between the two scenarios translate into differences in projected potential distributions. Almost all potential distributions proposed by CCSM conditions are nested within the MIROC projections. However, most areas not covered by CCSM but highlighted by MIROC are situated in nonanalogous climate space in CCSM, making predictions in these areas uncertain. As both scenarios represent snapshots of potential past range fluctuations assuming different environmental conditions, it may be reasonable to use an intersection of the predicted potential distributions as consensus, acknowledging the uncertainty of the predictions within extrapolation areas. This would suggest that the most likely occurrence areas of subsp. *spontaneum* about 21,000 yBP were situated within areas highlighted by the CCSM prediction, which comprise multiple smaller refugia within the eastern Mediterranean particularly in the Levant (also found by Russell et al. [2014]) as well as along the Zagros Mountains. Support for refugia in the Levant comes from archaeobotanical findings, as oldest wild barley remains were found at Ohalo II on the Sea of Galilee (Kislev et al. 1992), dating to 23,000 yBP. The earliest Neolithic site of Iran, Chogha Golan, where large amounts of wild barley were recently excavated (Riehl et al. 2013), lies well within the CCSM predicted refugia in the east. However, when interpreting the potential distributions as suggested under CCSM and MIROC conditions, it needs to be acknowledged that SDM projections highlight those areas providing suitable environmental conditions for the target species irrespective of biotic interactions and potential dispersal limitations (for a detailed discussion of pros and cons of SDM projections see, e.g., Elith and Leathwick 2009).

### Possible Scenario to Explain the Extant Distribution of Wild Barley

Taking into account the large diversity found within subsp. *spontaneum* at the analyzed loci, we assume that a large subsp. *spontaneum* population inhabited Southwest Asia for an extended time. We are, however, unable to infer the precise origin of this taxon, as the split within section *Hordeum*, that is, between *H. vulgare* and *H. bulbosum* lineages, dates back 5–6 Ma (Blattner 2006, 2009), and high extinction rates were proposed for Eurasian *Hordeum* during the Pleistocene (Jakob and Blattner 2006, 2010). This leaves the possibility open that other, now-extinct taxa occurred in the stem group of *H. vulgare*. From the distribution of genetic diversity, we assume that the initial population of subsp. *spontaneum* occurred in the Levant (including Cyprus) and southern Turkey, an area that provides large areas potentially suitable for subsp. *spontaneum* according to our SDM analyses. SDM also infers the Mediterranean coast of western Turkey as a potential distribution area, although it was assumed that subsp. *spontaneum* does not occur there naturally (Harlan and Zohary 1966). This Levant/Turkish population became separated most probably already before the Pleistocene into two separate genepools, building the stocks of today’s Western Cluster and Turkish Cluster. Both stocks might have consisted of many allopatric populations, thus maintaining high allele diversity. The high number of private alleles and singletons indicate that the Levant and southeastern Turkey populations rapidly regained large population sizes after their subdivision. Geographically structured genepools indicate that gene flow is low among these regions. Populations occurring in eastern Turkey and further east seem to be younger, and colonization of the east took place from the Turkish Cluster, as haplotypes are shared at nearly all loci between the Turkish and Eastern Cluster. SDM infers possible small ice-age refugia in the eastern area, particularly Iran, which are partly supported by archaeobotanical finds. However, we assume that either potential refugia were not occupied or that effective population sizes were so small that their genetic contribution to the current genepool of barley is negligible.

Although our phylogeographic data are clear regarding the division of subsp. *spontaneum* into three clusters, the extremely high haplotype diversity and its structure in the Western Cluster is hard to explain. The lack of major haplotypes, occupying the central positions in genealogical networks, and the high number of minor haplotypes are peculiar. Thus far, we could not envision a historical scenario that would convincingly explain such a pattern, as it involves particularly the loss of central haplotypes or the maintenance of haplotypes at low frequencies. It can be hypothesized that the higher *PPD-H1* allele richness and Nei’s gene diversity in the domesticated lines is probably due to the wider latitudinal origin of these genotypes and their different responsiveness to photoperiod. *ADH* genes have been related to flooding tolerance (e.g., Harberd and Edwards 1982), which can be considered as another important adaptive trait. Also *GAPDH* is intriguing, because two major haplotypes are found in both wild *H. spontaneum* and genebank *H. spontaneum*, which further differentiated in domesticated barleys. Apart from this*,* little information exists on probable selective pressure on the analyzed genes. Thus, assuming that the steep environmental gradient between coastal and desert habitats in the Levant might play a role in maintaining allele diversity by differential selection in small, isolated populations is speculative but the best explanation we have.

### Implications for Domestication Studies in Barley

At most analyzed loci, we found clear differences between the alleles occurring in barley and wild *H. spontaneum*. To explain this difference, we have to assume that due to differential selection under cultivation these two genepools drifted rapidly apart. This would, however, mean that most loci analyzed were influenced by disrupting selection during the ca. 11,000 years of barley cultivation. Another alternative is the possibility that already existing differences in the genepool of subsp. *spontaneum* were explored by early farmers, resulting in the inclusion of a certain ecotype in the crop, whereas the remaining type, that is, subsp. *spontaneum*, remained untouched forming extant populations of wild barley. Our study was, however, not designed to clarify the domestication history of barley.

A result important for further studies in *H. vulgare* is the comparatively high amount of individuals of wild barley found to be introgressed during ex situ conservation. Although introgression also occurs in nature, for example, when barley fields are in the vicinity of wild barley stands, the amount of gene flow is comparatively high for genebank materials. Therefore, we suggest treating the results from studies based mainly on genebank materials with some skepticism regarding their ability to correctly unravel evolutionary processes. Therefore, it appears necessary to collect a stock of accessions from wild barley populations for such research that is afterward maintained similar to obligate outbreeding taxa to prevent hybridization in genebanks.

## Supplementary Material

Supplementary text S1, figures S1–S5, and tables S1–S12 are available at *Genome Biology and Evolution* online (http://www.gbe.oxfordjournals.org/).

Supplementary Data
