# Supplementary material for: Evolutionary History of Wild Barley (Hordeum vulgare subsp. spontaneum) Analyzed Using Multilocus Sequence Data and Paleodistribution Modeling
Source: Genome Biol Evol. 2014 Feb 27;6(3):685–702. doi: 10.1093/gbe/evu047 (PMC3971598; doi:10.1093/gbe/evu047)
Supplement: Supplementary Data [file supp_6_3_685__index.html]

Evolutionary History of Wild Barley (Hordeum vulgare subsp. spontaneum) Analyzed Using Multilocus Sequence Data and Paleodistribution Modeling — Supplementary Data 

# Evolutionary History of Wild Barley (*Hordeum vulgare* subsp. *spontaneum*) Analyzed Using Multilocus Sequence Data and Paleodistribution Modeling

## Supplementary Data

files

**Files in this Data Supplement:**

- Supplementary Data - docx file
- Supplementary Data - xlsx file
- Supplementary Data - xlsx file
- Supplementary Data - xlsx file
- Supplementary Data - xlsx file
