# Supplementary material for: Evolutionary History of Wild Barley (Hordeum vulgare subsp. spontaneum) Analyzed Using Multilocus Sequence Data and Paleodistribution Modeling
Source: Genome Biol Evol. 2014 Feb 27;6(3):685–702. doi: 10.1093/gbe/evu047 (PMC3971598; doi:10.1093/gbe/evu047)
Supplement: Supplementary Data [file supp_evu047_Jakob_et_al_SI_GBE-140136_R_ED.docx]

## Evolutionary History of Wild Barley (*Hordeum vulgare* subsp. *spontaneum*) Analyzed Using Multilocus Sequence Data and Paleodistribution Modeling

Sabine S. Jakob^1^*, Dennis Rödder^2^, Jan O. Engler^2,3^, Salar Shaaf^1,4^, Hakan Özkan^5^, Frank R. Blattner^1,6^, Benjamin Kilian^1^*

^1^ Leibniz Institute of Plant Genetics and Crop Research (IPK), D-06466 Gatersleben, Germany

^2^ Zoologisches Forschungsmuseum Alexander Koenig, Adenauer Allee 160, D-53113 Bonn, Germany

^3^,Department of Wildlife Sciences, University of Göttingen, Büsgenweg 3, D-37077 Göttingen, Germany

^4^ Department of Agronomy and Plant Breeding, College of Agriculture and Natural Resources, Islamic Azad University, Sanandaj branch, 66169 Sanandaj, Iran

^5^ Department of Field Crops, Faculty of Agriculture, University of Çukurova, 01330 Adana, Turkey

^6^ German Centre for Integrative Biodiversity Research (iDiv) Halle-Jena-Leipzig, D-04103 Leipzig, Germany

***Corresponding authors:**

Sabine S. Jakob, Leibniz Institute of Plant Genetics and Crop Plant Research (IPK), Corrensstr. 3, D-06466 Gatersleben, Germany, Fax: +49-39482-5155, E-mail: **sabine.s.jakob@gmail.com**

Benjamin Kilian, Leibniz Institute of Plant Genetics and Crop Plant Research (IPK), Corrensstr. 3, D-06466 Gatersleben, Germany, Fax: +49-39482-5571, E-mail: **kilian@ipk-gatersleben.de**

**Data deposition:**

DNA sequences: GenBank accession no. KC661080 – KC661263.

Supplementary text, figures S1-S5, tables S1-S12 are available at *Genome Biology and Evolution* online (<http://www.gbe.oxfordjournals.org/>). The Supplementary information has been arranged into the following sections:

**Supplementary text**

Extended methods ……………………………………..………………… page 3

**Supplementary figures**

Supplementary figure S1 ……………………….…………..…………… page 26

Supplementary figure S2 …………….………….……………………… page 28

Supplementary figure S3 ……………..…….…………………………… page 30

Supplementary figure S4 …………………...…………………………… page 32

Supplementary figure S5 …………………...…………………………… page 33

**Supplementary tables**

Supplementary table S1 …………………….…………………………… page 34

Supplementary table S2 …………………….…………………………… page 35

Supplementary table S3 ……………………….………………………… page 36

Supplementary table S4 ……………………….………………………… page 37

Supplementary table S5 ……………………….………………………… page 38

Supplementary table S6 ………………………….……………………… page 39

Supplementary table S7 ………………………….……………………… page 40

Supplementary table S8 ……………………………….………………… page 41

Supplementary table S9 ……………………………….………………… page 42

Supplementary table S10 ……………………...………………………… page 43

Supplementary table S11 ………………………...……………………… page 44

Supplementary table S12 ………………………...……………………… page 45

Extended methods

### DNA amplification, sequencing and sequence analysis

For the *PPD-H1* locus on chromosome 2HS (Turner et al. 2005; Hemming et al. 2008) the Primer3 online software (primer3_www.cgi v 0.2, Whitehead Institute for Biomedical Research, Cambridge, UK) (Rozen and Skaletsky 2000) was used to design primers, which amplified the last two exons and the last three introns of *PPD-H1*: PP05 (forward) 5’-GTGCAAAGCATAATATCAGTGTCC-3’ and PP04 (reverse) 5’-GGCCAAAGACACAAGAATCAG-3’. The total amplified fragment size of *PPD-H1* for cultivar ‘Morex’ was 1013 base pairs (bp), from which 888 bp were considered for the multiple sequence alignment. PCR was performed with a GeneAmp 9700 PCR System (PE Biosystems) with the program: 94 °C for 3 min, 35 cycles of 94 °C for 30 sec, 60.5 °C for 40 sec, and 72 °C for 2 min, followed by a post treatment at 72 °C for 8 min. The reactions were carried out with 1.5 U *Taq* polymerase (Qiagen) in the supplied reaction buffer, 0.2 mM of each dNTP, 50 pmol of each primer and about 20 ng of total DNA in a final reaction volume of 50 µl. Amplicons for all loci were checked on agarose gels, purified using Nucleofast 96 Spin Plates (Macherey-Nagel) and sequenced directly for both strands on an ABI Prism 3730xL sequencer using the respective BigDye terminator chemistry (Applied Biosystems). DNA sequences were checked and manually edited in Chromas Pro (Technelysium Pty Ltd) and sequence alignments generated with BioEdit version 7.0.9.0 (Hall 1999). Allelic variants found only once (singletons) were confirmed by three independent amplifications and sequencing. Length variation at microsatellite motives [one four- (CTGT/GCAG) and one dinucleotide (GT/AC) motive in *DHN9*, two trinucleotide repeats in *PPD-H1* (AGC/GCT and CTG/CAG)] were excluded from the analyses due to their uncertain homology. Each insertion/deletion (indel) was considered as a single mutation event, and all indels were therefore coded as single positions. Identical sequences were grouped into haplotypes (HT). In addition to the sequences produced within this study we included the sequences of Morrell et al. (2003) and Kilian et al. (2006) in multiple sequence alignments. The observed haplotypes are listed in supplementary tables S2 and S3. Among 415 accessions, 381 were homozygous at all loci sampled; only nine individuals (7x ‘wild *H. spontaneum*’; 2x ‘genebank *H. spontaneum*’) were heterozygous at more than one locus. Thus 98.3% of all loci were homozygous. No haplotype information was obtained at 38.5 loci (1.3%).

***Network reconstruction***

Neighbor-Net planar graphs of Hamming distances (uncorrected *p*-distance) between all 123 ‘wild *H. spontaneum*’ samples were constructed based on haplotypes at seven sequenced nuclear loci using SplitsTree 4.1 (Huson and Bryant 2006). Haplotypes were equally weighted and coded for each individual at all loci in a binary matrix for presence (1) and absence (0). Subsequently the matrices of all loci were concatenated.

***Structure Analysis***

Bayesian cluster assignment analyses were performed to infer the spatial structure in the genetic data of 123 ‘wild *H. spontaneum*’ individuals using Structure 2.3.3 (Pritchard et al. 2000, 2004; Falush et al. 2003). To infer the number of genetic groups (*K*) in our sample of ‘wild *H. spontaneum*’ we used a multistep approach (after several trial runs). The first step of the analysis consisted of estimating *K*. Twenty independent runs of *K* from 1 to 10 with 1,000,000 MCMC iterations and a burn-in period of 500,000 were performed, using the model with correlated allele frequencies and assuming admixture. The initial value for alpha, the Dirichlet parameter for degree of admixture, was set to 1. To infer the appropriate number of *K*, Structure Harvester (Earl & von Holdt 2012; http://taylor0.biology.ucla.edu/structureHarvester/index.php) was used. In a second step, after the inference of *K*, the Structure procedure was repeated with a fixed *K* and 10 independent runs with 500,000 MCMC iterations and a burn-in period of 250,000. To finally assign each individual to the cluster for which it showed the highest percentage of membership (q) the program Clumpp (Jakobsson and Rosenberg 2007) was applied. An individual has been assigned to a certain cluster if its q value was higher than 0.6. Individuals with maximum inferred ancestry <0.6 were not assigned to any cluster (“admixed”, as they fall in between several clusters) (supplementary fig. S1, supplementary table S4).

From an initial set of assignment and genealogical analyses we concluded that a large number of ‘genebank *H. spontaneum*’ accessions showed signs of admixture which we did not observe in ‘wild *H. spontaneum*’. To avoid wrong cluster assignments for subsp. *spontaneum* individuals due to such hybridization traces, the 152 individuals of ‘genebank *H. spontaneum*’ as well as the 140 domesticated barleys have been separately assigned to the groups already inferred by Structure and thus using prior population information from ‘wild *H. spontaneum*’ to assist clustering in Structure. The MIGRPRIOR (migration prior) was set to 0.1 to allow for some misclassification. The burn-in period consisted of 250,000 and the MCMC of 500,000 iterations (supplementary fig. S1, supplementary table S4).

**CLUMMP seetings**

CLUMPP_paramfile_K3.txt:

# This is the file that sets the parameters for the program CLUMPP, version 1.1

# Everything after "#" will be ignored by the program. Parameters are:

# K, C, R, M, W, GREEDY_OPTION, REPEATS, PERMUTATIONFILE, PRINT_PERMUTED_DATA,

# PERMUTED_DATAFILE, PRINT_EVERY_PERM and EVERY_PERMFILE.

# All parameter names shall be followed by at least one blank space and then

# the parameter-value.

# --------------- Main parameters ---------------------------------------------

DATATYPE 0 # The type of data to be read in.

# 0 = individual data in the file

# specified by INDFILE, 1 = population

# data in the file specified by

# POPFILE.

INDFILE K3.indfile # The name of the individual datafile.

# Required if DATATYPE = 0.

POPFILE K3.popfile # The name of the population datafile.

# Required if DATATYPE = 1.

OUTFILE K3.outfile # The average cluster membership

# coefficients across the permuted runs

# are printed here.

MISCFILE K3.miscfile # The parameters used and a summary of

# the results are printed here.

K 3 # Number of clusters.

C 123 # Number of individuals or populations.

R 20 # Number of runs.

M 2 # Method to be used (1 = FullSearch,

# 2 = Greedy, 3 = LargeKGreedy).

W 1 # Weight by the number of individuals

# in each population as specified in

# the datafile (1 if yes, 0 if no).

S 2 # Pairwise matrix similarity statistic

# to be used. 1 = G, 2 = G'.

# - Additional options for the Greedy and LargeKGreedy algorithm (M = 2 or 3) -

GREEDY_OPTION 2 # 1 = All possible input orders,

# 2 = random input orders,

# 3 = pre-specified input orders.

REPEATS 1000 # If GREEDY_OPTION = 2, then REPEATS

# determines the number of random input

# orders to be tested. If GREEDY_OPTION

# = 3, then REPEATS is the number of

# input orders in PERMUTATIONFILE.

PERMUTATIONFILE K3.permutationfile # The permutations of the runs in

# PERMUTATIONFILE will be used, if

# GREEDY_OPTION = 3.

# --------------- Optional outputs --------------------------------------------

PRINT_PERMUTED_DATA 1 # Print the permuted data (clusters) in

# INDFILE or POPFILE to

# PERMUTED_DATAFILE (0 = don't print,

# 1 = print into one file, 2 = print

# into separate files for each run).

PERMUTED_DATAFILE K3.perm_datafile # The permuted data (clusters) will be

# printed to this file (if

# PRINT_PERMUTED_DATA = 2, several

# files with the extensions "_1" to

# "_R" will be created).

PRINT_EVERY_PERM 0 # Print every tested permutation of the

# runs and the corresponding value of

# SSC to a file specified by

# EVERY_PERMFILE (0 = don't print,

# 1 = print).

# Note that printing may result in a

# very large file.

EVERY_PERMFILE K3.every_permfile # Every tested permutation of the runs

# and the corresponding SSC will be

# printed here.

PRINT_RANDOM_INPUTORDER 0 # Print random input orders of runs to

# RANDOM_INPUTORDER (0 = don't print,

# 1 = print). This option is only

# available if GREEDY_OPTION = 2.

RANDOM_INPUTORDERFILE K3.random_inputorderfile # Every random input order

# of the runs (generated by CLUMPP if

# GREEDY_OPTION = 2) will be printed

# here.

# --------------- Advanced options --------------------------------------------

OVERRIDE_WARNINGS 0 # This option allows the user to

# override non-crucial warnings from

# the program (0 allow warnings, 1 do

# not issue non-crucial warnings).

ORDER_BY_RUN 1 # Permute the clusters of the output

# files by the specified run. (0 to

# not specify a run, 1 to R specifies

# a run in the INDFILE or POPFILE).

# --------------- Additional comments -----------------------------------------

# The term ''permutation'' is used in two different contexts, permutations of

# membership coefficients, or clusters, and permutations of runs.

# For example, if the datafile has has data A B C D E (each letter indicates a

# column corresponding to a cluster), then permutation 3 2 5 1 4 of the

# clusters means C B E A D.

# Permutation 4 1 2 3 of runs 1-4 would mean start with run 4, then run 1, then

# run 2, and then run 3.

# --------------- Command line arguments --------------------------------------

# -i INDFILE

# -p POPFILE

# -o OUTFILE

# -j MISCFILE

# -k K

# -c C

# -r R

# -m M

# -w W

# -s S

# -----------------------------------------------------------------------------

CLUMPP_Settings_K3.miscfile.txt:

Using the parameter settings in the file:

''paramfile''

Parameter settings

----------------------- Main parameters -------------------------

DATATYPE = 0

INDFILE = K3.indfile

POPFILE =

OUTFILE = K3.outfile

MISCFILE = K3.miscfile

K = 3

C = 123

R = 20

M = 2

W = 1

S = 2

- Additional options for the Greedy and LargeKGreedy algorithms -

GREEDY_OPTION = 2

REPEATS = 1000

PERMUTATIONFILE =

----------------------- Optional outputs ------------------------

PRINT_PERMUTED_DATA = 1

PERMUTED_DATAFILE = K3.perm_datafile

PRINT_EVERY_PERM = 0

EVERY_PERMFILE =

PRINT_RANDOM_INPUTORDER = 0

RANDOM_INPUTORDERFILE =

----------------------- Advanced options ------------------------

OVERRIDE_WARNINGS = 0

ORDER_BY_RUN = 1

In total, 1000 conFigurations of runs and clusters will be tested.

Results

------------------------------

The highest value of H' is: 0.84464343941208

The list of permutations of the clusters that produces that

H' value is (runs are listed sequentially on separate rows)

1 2 3

3 2 1

2 3 1

3 1 2

3 1 2

1 3 2

2 1 3

2 3 1

2 1 3

3 2 1

2 3 1

1 3 2

1 2 3

3 2 1

3 2 1

2 1 3

2 1 3

1 2 3

1 3 2

1 2 3

The pairwise G' values for each pair of runs where the clusters

of each run are permuted according to the list of permutations above

1.0000 0.9932 0.9928 0.5627 0.9939 0.9921 0.9928 0.5776 0.9926 0.9935 0.9935 0.5288 0.9945 0.9932 0.9963 0.9921 0.9922 0.5717 0.9930 0.9924

0.9932 1.0000 0.9956 0.5623 0.9955 0.9962 0.9961 0.5773 0.9963 0.9961 0.9972 0.5284 0.9888 0.9958 0.9926 0.9958 0.9949 0.5713 0.9965 0.9971

0.9928 0.9956 1.0000 0.5629 0.9942 0.9938 0.9961 0.5780 0.9960 0.9964 0.9964 0.5289 0.9888 0.9969 0.9925 0.9935 0.9967 0.5719 0.9963 0.9947

0.5627 0.5623 0.5629 1.0000 0.5622 0.5630 0.5631 0.8896 0.5631 0.5635 0.5622 0.9038 0.5621 0.5627 0.5617 0.5620 0.5626 0.9585 0.5623 0.5628

0.9939 0.9955 0.9942 0.5622 1.0000 0.9943 0.9951 0.5771 0.9951 0.9957 0.9960 0.5283 0.9899 0.9950 0.9928 0.9943 0.9942 0.5712 0.9949 0.9948

0.9921 0.9962 0.9938 0.5630 0.9943 1.0000 0.9952 0.5779 0.9952 0.9944 0.9951 0.5290 0.9878 0.9939 0.9910 0.9955 0.9927 0.5719 0.9945 0.9967

0.9928 0.9961 0.9961 0.5631 0.9951 0.9952 1.0000 0.5781 0.9967 0.9972 0.9963 0.5291 0.9889 0.9960 0.9924 0.9938 0.9964 0.5721 0.9958 0.9947

0.5776 0.5773 0.5780 0.8896 0.5771 0.5779 0.5781 1.0000 0.5781 0.5784 0.5771 0.7935 0.5769 0.5778 0.5767 0.5771 0.5776 0.9304 0.5773 0.5778

0.9926 0.9963 0.9960 0.5631 0.9951 0.9952 0.9967 0.5781 1.0000 0.9962 0.9961 0.5291 0.9889 0.9963 0.9926 0.9940 0.9957 0.5721 0.9959 0.9951

0.9935 0.9961 0.9964 0.5635 0.9957 0.9944 0.9972 0.5784 0.9962 1.0000 0.9962 0.5295 0.9896 0.9959 0.9925 0.9934 0.9960 0.5724 0.9956 0.9949

0.9935 0.9972 0.9964 0.5622 0.9960 0.9951 0.9963 0.5771 0.9961 0.9962 1.0000 0.5283 0.9889 0.9974 0.9930 0.9962 0.9961 0.5712 0.9983 0.9965

0.5288 0.5284 0.5289 0.9038 0.5283 0.5290 0.5291 0.7935 0.5291 0.5295 0.5283 1.0000 0.5283 0.5287 0.5278 0.5280 0.5285 0.8627 0.5283 0.5288

0.9945 0.9888 0.9888 0.5621 0.9899 0.9878 0.9889 0.5769 0.9889 0.9896 0.9889 0.5283 1.0000 0.9888 0.9941 0.9872 0.9884 0.5710 0.9883 0.9877

0.9932 0.9958 0.9969 0.5627 0.9950 0.9939 0.9960 0.5778 0.9963 0.9959 0.9974 0.5287 0.9888 1.0000 0.9932 0.9948 0.9968 0.5717 0.9975 0.9949

0.9963 0.9926 0.9925 0.5617 0.9928 0.9910 0.9924 0.5767 0.9926 0.9925 0.9930 0.5278 0.9941 0.9932 1.0000 0.9912 0.9926 0.5707 0.9927 0.9912

0.9921 0.9958 0.9935 0.5620 0.9943 0.9955 0.9938 0.5771 0.9940 0.9934 0.9962 0.5280 0.9872 0.9948 0.9912 1.0000 0.9929 0.5710 0.9961 0.9969

0.9922 0.9949 0.9967 0.5626 0.9942 0.9927 0.9964 0.5776 0.9957 0.9960 0.9961 0.5285 0.9884 0.9968 0.9926 0.9929 1.0000 0.5716 0.9960 0.9934

0.5717 0.5713 0.5719 0.9585 0.5712 0.5719 0.5721 0.9304 0.5721 0.5724 0.5712 0.8627 0.5710 0.5717 0.5707 0.5710 0.5716 1.0000 0.5713 0.5718

0.9930 0.9965 0.9963 0.5623 0.9949 0.9945 0.9958 0.5773 0.9959 0.9956 0.9983 0.5283 0.9883 0.9975 0.9927 0.9961 0.9960 0.5713 1.0000 0.9961

0.9924 0.9971 0.9947 0.5628 0.9948 0.9967 0.9947 0.5778 0.9951 0.9949 0.9965 0.5288 0.9877 0.9949 0.9912 0.9969 0.9934 0.5718 0.9961 1.0000

Result_summary_STRUCTURE harvester.html"

| **K** | **Reps** | **Mean LnP(K)** | **Stdev LnP(K)** | **Ln'(K)** | **\|Ln''(K)\|** | **Delta K** |
| --- | --- | --- | --- | --- | --- | --- |
| 1 | 20 | -2812.850000 | 0.392696 | — | — | — |
| 2 | 20 | -2483.935000 | 13.090908 | 328.915000 | 84.905000 | 6.485799 |
| 3 | 20 | -2239.925000 | 10.270493 | 244.010000 | 81.580000 | 7.943144 |
| 4 | 20 | -2077.495000 | 11.959690 | 162.430000 | 61.655000 | 5.155234 |
| 5 | 20 | -1976.720000 | 14.951979 | 100.775000 | 12.065000 | 0.806917 |
| 6 | 20 | -1863.880000 | 6.838790 | 112.840000 | 27.390000 | 4.005094 |
| 7 | 20 | -1778.430000 | 23.835005 | 85.450000 | 24.020000 | 1.007761 |
| 8 | 20 | -1717.000000 | 63.732987 | 61.430000 | 19.040000 | 0.298746 |
| 9 | 20 | -1636.530000 | 5.435952 | 80.470000 | 20.190000 | 3.714161 |
| 10 | 20 | -1576.250000 | 5.644886 | 60.280000 | — | — |

## Raw STRUCTURE output

| **File name** | **Run #** | **K** | **Est. Ln prob. of data** | **Mean value of Ln likelihood** | **Variance of Ln likelihood** |
| --- | --- | --- | --- | --- | --- |
| Resultsadmix_corel_500_1000_run_1_f | 1 | 1 | -2813.0 | -2787.1 | 52.0 |
| Resultsadmix_corel_500_1000_run_10_f | 10 | 1 | -2813.3 | -2787.0 | 52.6 |
| Resultsadmix_corel_500_1000_run_11_f | 11 | 1 | -2813.1 | -2787.0 | 52.2 |
| Resultsadmix_corel_500_1000_run_12_f | 12 | 1 | -2813.4 | -2787.1 | 52.7 |
| Resultsadmix_corel_500_1000_run_13_f | 13 | 1 | -2813.0 | -2787.0 | 51.8 |
| Resultsadmix_corel_500_1000_run_14_f | 14 | 1 | -2813.4 | -2787.0 | 52.7 |
| Resultsadmix_corel_500_1000_run_15_f | 15 | 1 | -2812.7 | -2787.0 | 51.5 |
| Resultsadmix_corel_500_1000_run_16_f | 16 | 1 | -2811.9 | -2786.9 | 50.0 |
| Resultsadmix_corel_500_1000_run_17_f | 17 | 1 | -2813.1 | -2787.2 | 51.8 |
| Resultsadmix_corel_500_1000_run_18_f | 18 | 1 | -2812.6 | -2787.1 | 51.0 |
| Resultsadmix_corel_500_1000_run_19_f | 19 | 1 | -2813.2 | -2787.2 | 52.0 |
| Resultsadmix_corel_500_1000_run_2_f | 2 | 1 | -2812.9 | -2786.9 | 52.0 |
| Resultsadmix_corel_500_1000_run_20_f | 20 | 1 | -2812.4 | -2787.1 | 50.7 |
| Resultsadmix_corel_500_1000_run_3_f | 3 | 1 | -2812.5 | -2786.9 | 51.3 |
| Resultsadmix_corel_500_1000_run_4_f | 4 | 1 | -2812.8 | -2787.3 | 51.0 |
| Resultsadmix_corel_500_1000_run_5_f | 5 | 1 | -2813.1 | -2786.9 | 52.4 |
| Resultsadmix_corel_500_1000_run_6_f | 6 | 1 | -2812.5 | -2787.0 | 50.9 |
| Resultsadmix_corel_500_1000_run_7_f | 7 | 1 | -2812.4 | -2787.1 | 50.7 |
| Resultsadmix_corel_500_1000_run_8_f | 8 | 1 | -2813.1 | -2787.3 | 51.6 |
| Resultsadmix_corel_500_1000_run_9_f | 9 | 1 | -2812.6 | -2787.0 | 51.3 |
| Resultsadmix_corel_500_1000_run_21_f | 21 | 2 | -2472.7 | -2383.9 | 177.5 |
| Resultsadmix_corel_500_1000_run_22_f | 22 | 2 | -2474.4 | -2383.7 | 181.5 |
| Resultsadmix_corel_500_1000_run_23_f | 23 | 2 | -2474.2 | -2383.8 | 180.8 |
| Resultsadmix_corel_500_1000_run_24_f | 24 | 2 | -2478.8 | -2403.2 | 151.2 |
| Resultsadmix_corel_500_1000_run_25_f | 25 | 2 | -2475.9 | -2384.5 | 182.7 |
| Resultsadmix_corel_500_1000_run_26_f | 26 | 2 | -2477.2 | -2384.9 | 184.6 |
| Resultsadmix_corel_500_1000_run_27_f | 27 | 2 | -2480.5 | -2384.5 | 192.0 |
| Resultsadmix_corel_500_1000_run_28_f | 28 | 2 | -2484.3 | -2384.7 | 199.2 |
| Resultsadmix_corel_500_1000_run_29_f | 29 | 2 | -2477.1 | -2402.1 | 150.1 |
| Resultsadmix_corel_500_1000_run_30_f | 30 | 2 | -2480.4 | -2384.7 | 191.5 |
| Resultsadmix_corel_500_1000_run_31_f | 31 | 2 | -2479.9 | -2403.3 | 153.2 |
| Resultsadmix_corel_500_1000_run_32_f | 32 | 2 | -2518.4 | -2389.2 | 258.5 |
| Resultsadmix_corel_500_1000_run_33_f | 33 | 2 | -2488.0 | -2385.9 | 204.2 |
| Resultsadmix_corel_500_1000_run_34_f | 34 | 2 | -2502.1 | -2387.2 | 229.7 |
| Resultsadmix_corel_500_1000_run_35_f | 35 | 2 | -2477.8 | -2384.6 | 186.4 |
| Resultsadmix_corel_500_1000_run_36_f | 36 | 2 | -2516.5 | -2387.9 | 257.2 |
| Resultsadmix_corel_500_1000_run_37_f | 37 | 2 | -2478.2 | -2384.9 | 186.4 |
| Resultsadmix_corel_500_1000_run_38_f | 38 | 2 | -2482.5 | -2385.3 | 194.5 |
| Resultsadmix_corel_500_1000_run_39_f | 39 | 2 | -2483.1 | -2385.1 | 196.1 |
| Resultsadmix_corel_500_1000_run_40_f | 40 | 2 | -2476.7 | -2402.9 | 147.6 |
| Resultsadmix_corel_500_1000_run_41_f | 41 | 3 | -2242.9 | -2145.8 | 194.2 |
| Resultsadmix_corel_500_1000_run_42_f | 42 | 3 | -2242.8 | -2145.5 | 194.6 |
| Resultsadmix_corel_500_1000_run_43_f | 43 | 3 | -2244.4 | -2145.7 | 197.4 |
| Resultsadmix_corel_500_1000_run_44_f | 44 | 3 | -2229.0 | -2124.6 | 208.9 |
| Resultsadmix_corel_500_1000_run_45_f | 45 | 3 | -2258.0 | -2146.7 | 222.6 |
| Resultsadmix_corel_500_1000_run_46_f | 46 | 3 | -2240.4 | -2145.0 | 190.8 |
| Resultsadmix_corel_500_1000_run_47_f | 47 | 3 | -2240.6 | -2145.0 | 191.3 |
| Resultsadmix_corel_500_1000_run_48_f | 48 | 3 | -2217.1 | -2130.4 | 173.5 |
| Resultsadmix_corel_500_1000_run_49_f | 49 | 3 | -2244.6 | -2145.6 | 198.0 |
| Resultsadmix_corel_500_1000_run_50_f | 50 | 3 | -2246.6 | -2145.9 | 201.6 |
| Resultsadmix_corel_500_1000_run_51_f | 51 | 3 | -2243.9 | -2145.7 | 196.4 |
| Resultsadmix_corel_500_1000_run_52_f | 52 | 3 | -2213.3 | -2119.6 | 187.3 |
| Resultsadmix_corel_500_1000_run_53_f | 53 | 3 | -2243.2 | -2145.5 | 195.5 |
| Resultsadmix_corel_500_1000_run_54_f | 54 | 3 | -2242.0 | -2145.5 | 193.0 |
| Resultsadmix_corel_500_1000_run_55_f | 55 | 3 | -2241.6 | -2145.2 | 192.7 |
| Resultsadmix_corel_500_1000_run_56_f | 56 | 3 | -2247.2 | -2145.9 | 202.6 |
| Resultsadmix_corel_500_1000_run_57_f | 57 | 3 | -2246.2 | -2145.8 | 200.7 |
| Resultsadmix_corel_500_1000_run_58_f | 58 | 3 | -2230.9 | -2127.3 | 207.3 |
| Resultsadmix_corel_500_1000_run_59_f | 59 | 3 | -2239.8 | -2145.3 | 188.9 |
| Resultsadmix_corel_500_1000_run_60_f | 60 | 3 | -2244.0 | -2145.8 | 196.4 |
| Resultsadmix_corel_500_1000_run_61_f | 61 | 4 | -2078.0 | -1949.8 | 256.4 |
| Resultsadmix_corel_500_1000_run_62_f | 62 | 4 | -2075.5 | -1949.7 | 251.6 |
| Resultsadmix_corel_500_1000_run_63_f | 63 | 4 | -2071.9 | -1950.1 | 243.5 |
| Resultsadmix_corel_500_1000_run_64_f | 64 | 4 | -2084.3 | -1950.7 | 267.2 |
| Resultsadmix_corel_500_1000_run_65_f | 65 | 4 | -2071.2 | -1949.0 | 244.3 |
| Resultsadmix_corel_500_1000_run_66_f | 66 | 4 | -2071.6 | -1949.3 | 244.7 |
| Resultsadmix_corel_500_1000_run_67_f | 67 | 4 | -2075.8 | -1950.0 | 251.7 |
| Resultsadmix_corel_500_1000_run_68_f | 68 | 4 | -2070.9 | -1949.6 | 242.7 |
| Resultsadmix_corel_500_1000_run_69_f | 69 | 4 | -2071.7 | -1949.1 | 245.2 |
| Resultsadmix_corel_500_1000_run_70_f | 70 | 4 | -2071.5 | -1949.4 | 244.2 |
| Resultsadmix_corel_500_1000_run_71_f | 71 | 4 | -2076.8 | -1949.9 | 253.9 |
| Resultsadmix_corel_500_1000_run_72_f | 72 | 4 | -2075.5 | -1950.0 | 251.0 |
| Resultsadmix_corel_500_1000_run_73_f | 73 | 4 | -2125.8 | -1954.7 | 342.3 |
| Resultsadmix_corel_500_1000_run_74_f | 74 | 4 | -2077.7 | -1950.2 | 255.0 |
| Resultsadmix_corel_500_1000_run_75_f | 75 | 4 | -2075.2 | -1949.3 | 251.8 |
| Resultsadmix_corel_500_1000_run_76_f | 76 | 4 | -2075.9 | -1949.8 | 252.2 |
| Resultsadmix_corel_500_1000_run_77_f | 77 | 4 | -2081.2 | -1950.3 | 261.8 |
| Resultsadmix_corel_500_1000_run_78_f | 78 | 4 | -2077.1 | -1949.7 | 254.6 |
| Resultsadmix_corel_500_1000_run_79_f | 79 | 4 | -2072.8 | -1949.6 | 246.4 |
| Resultsadmix_corel_500_1000_run_80_f | 80 | 4 | -2069.5 | -1948.7 | 241.6 |
| Resultsadmix_corel_500_1000_run_100_f | 100 | 5 | -1979.3 | -1829.8 | 298.9 |
| Resultsadmix_corel_500_1000_run_81_f | 81 | 5 | -1974.2 | -1829.3 | 289.7 |
| Resultsadmix_corel_500_1000_run_82_f | 82 | 5 | -1997.8 | -1832.2 | 331.3 |
| Resultsadmix_corel_500_1000_run_83_f | 83 | 5 | -1981.5 | -1829.7 | 303.7 |
| Resultsadmix_corel_500_1000_run_84_f | 84 | 5 | -1977.9 | -1830.0 | 295.8 |
| Resultsadmix_corel_500_1000_run_85_f | 85 | 5 | -1990.1 | -1830.7 | 318.8 |
| Resultsadmix_corel_500_1000_run_86_f | 86 | 5 | -1981.2 | -1830.0 | 302.4 |
| Resultsadmix_corel_500_1000_run_87_f | 87 | 5 | -1948.4 | -1820.6 | 255.7 |
| Resultsadmix_corel_500_1000_run_88_f | 88 | 5 | -1954.5 | -1821.3 | 266.4 |
| Resultsadmix_corel_500_1000_run_89_f | 89 | 5 | -1976.6 | -1829.0 | 295.2 |
| Resultsadmix_corel_500_1000_run_90_f | 90 | 5 | -1955.3 | -1822.3 | 266.2 |
| Resultsadmix_corel_500_1000_run_91_f | 91 | 5 | -1981.3 | -1829.8 | 303.0 |
| Resultsadmix_corel_500_1000_run_92_f | 92 | 5 | -1977.3 | -1830.0 | 294.6 |
| Resultsadmix_corel_500_1000_run_93_f | 93 | 5 | -1987.0 | -1830.2 | 313.6 |
| Resultsadmix_corel_500_1000_run_94_f | 94 | 5 | -1946.4 | -1820.4 | 252.0 |
| Resultsadmix_corel_500_1000_run_95_f | 95 | 5 | -1981.5 | -1830.4 | 302.2 |
| Resultsadmix_corel_500_1000_run_96_f | 96 | 5 | -1992.5 | -1831.8 | 321.5 |
| Resultsadmix_corel_500_1000_run_97_f | 97 | 5 | -1990.9 | -1828.1 | 325.7 |
| Resultsadmix_corel_500_1000_run_98_f | 98 | 5 | -1968.8 | -1821.8 | 293.9 |
| Resultsadmix_corel_500_1000_run_99_f | 99 | 5 | -1991.9 | -1830.5 | 322.9 |
| Resultsadmix_corel_500_1000_run_101_f | 101 | 6 | -1859.6 | -1706.8 | 305.6 |
| Resultsadmix_corel_500_1000_run_102_f | 102 | 6 | -1862.3 | -1707.3 | 309.9 |
| Resultsadmix_corel_500_1000_run_103_f | 103 | 6 | -1886.6 | -1708.8 | 355.4 |
| Resultsadmix_corel_500_1000_run_104_f | 104 | 6 | -1865.4 | -1708.5 | 313.8 |
| Resultsadmix_corel_500_1000_run_105_f | 105 | 6 | -1861.7 | -1706.8 | 309.8 |
| Resultsadmix_corel_500_1000_run_106_f | 106 | 6 | -1868.3 | -1708.7 | 319.2 |
| Resultsadmix_corel_500_1000_run_107_f | 107 | 6 | -1861.6 | -1707.3 | 308.5 |
| Resultsadmix_corel_500_1000_run_108_f | 108 | 6 | -1859.8 | -1706.8 | 305.9 |
| Resultsadmix_corel_500_1000_run_109_f | 109 | 6 | -1863.2 | -1707.5 | 311.3 |
| Resultsadmix_corel_500_1000_run_110_f | 110 | 6 | -1865.8 | -1707.5 | 316.6 |
| Resultsadmix_corel_500_1000_run_111_f | 111 | 6 | -1864.2 | -1707.8 | 312.9 |
| Resultsadmix_corel_500_1000_run_112_f | 112 | 6 | -1861.5 | -1707.6 | 307.7 |
| Resultsadmix_corel_500_1000_run_113_f | 113 | 6 | -1860.2 | -1706.8 | 306.8 |
| Resultsadmix_corel_500_1000_run_114_f | 114 | 6 | -1862.9 | -1706.9 | 312.0 |
| Resultsadmix_corel_500_1000_run_115_f | 115 | 6 | -1869.8 | -1708.0 | 323.6 |
| Resultsadmix_corel_500_1000_run_116_f | 116 | 6 | -1859.6 | -1707.8 | 303.5 |
| Resultsadmix_corel_500_1000_run_117_f | 117 | 6 | -1854.8 | -1706.6 | 296.4 |
| Resultsadmix_corel_500_1000_run_118_f | 118 | 6 | -1872.7 | -1707.8 | 329.7 |
| Resultsadmix_corel_500_1000_run_119_f | 119 | 6 | -1856.2 | -1715.6 | 281.2 |
| Resultsadmix_corel_500_1000_run_120_f | 120 | 6 | -1861.4 | -1707.0 | 308.8 |
| Resultsadmix_corel_500_1000_run_121_f | 121 | 7 | -1788.8 | -1607.6 | 362.3 |
| Resultsadmix_corel_500_1000_run_122_f | 122 | 7 | -1773.3 | -1606.9 | 332.8 |
| Resultsadmix_corel_500_1000_run_123_f | 123 | 7 | -1797.6 | -1609.7 | 376.0 |
| Resultsadmix_corel_500_1000_run_124_f | 124 | 7 | -1762.9 | -1604.7 | 316.5 |
| Resultsadmix_corel_500_1000_run_125_f | 125 | 7 | -1771.9 | -1606.0 | 331.8 |
| Resultsadmix_corel_500_1000_run_126_f | 126 | 7 | -1769.1 | -1606.2 | 325.9 |
| Resultsadmix_corel_500_1000_run_127_f | 127 | 7 | -1768.0 | -1606.4 | 323.1 |
| Resultsadmix_corel_500_1000_run_128_f | 128 | 7 | -1769.4 | -1606.5 | 325.8 |
| Resultsadmix_corel_500_1000_run_129_f | 129 | 7 | -1771.5 | -1606.5 | 330.0 |
| Resultsadmix_corel_500_1000_run_130_f | 130 | 7 | -1789.2 | -1608.6 | 361.3 |
| Resultsadmix_corel_500_1000_run_131_f | 131 | 7 | -1767.6 | -1605.4 | 324.5 |
| Resultsadmix_corel_500_1000_run_132_f | 132 | 7 | -1769.9 | -1606.2 | 327.4 |
| Resultsadmix_corel_500_1000_run_133_f | 133 | 7 | -1768.1 | -1605.1 | 326.0 |
| Resultsadmix_corel_500_1000_run_134_f | 134 | 7 | -1765.4 | -1607.8 | 315.2 |
| Resultsadmix_corel_500_1000_run_135_f | 135 | 7 | -1770.4 | -1607.1 | 326.6 |
| Resultsadmix_corel_500_1000_run_136_f | 136 | 7 | -1872.5 | -1619.3 | 506.5 |
| Resultsadmix_corel_500_1000_run_137_f | 137 | 7 | -1766.5 | -1605.6 | 321.8 |
| Resultsadmix_corel_500_1000_run_138_f | 138 | 7 | -1774.4 | -1607.3 | 334.1 |
| Resultsadmix_corel_500_1000_run_139_f | 139 | 7 | -1773.1 | -1607.3 | 331.5 |
| Resultsadmix_corel_500_1000_run_140_f | 140 | 7 | -1779.0 | -1606.4 | 345.2 |
| Resultsadmix_corel_500_1000_run_141_f | 141 | 8 | -1708.9 | -1529.8 | 358.2 |
| Resultsadmix_corel_500_1000_run_142_f | 142 | 8 | -1708.6 | -1537.6 | 341.9 |
| Resultsadmix_corel_500_1000_run_143_f | 143 | 8 | -1704.2 | -1530.2 | 348.1 |
| Resultsadmix_corel_500_1000_run_144_f | 144 | 8 | -1696.6 | -1530.2 | 332.9 |
| Resultsadmix_corel_500_1000_run_145_f | 145 | 8 | -1704.5 | -1531.9 | 345.2 |
| Resultsadmix_corel_500_1000_run_146_f | 146 | 8 | -1710.7 | -1539.3 | 342.8 |
| Resultsadmix_corel_500_1000_run_147_f | 147 | 8 | -1707.6 | -1537.7 | 340.0 |
| Resultsadmix_corel_500_1000_run_148_f | 148 | 8 | -1703.4 | -1528.5 | 349.8 |
| Resultsadmix_corel_500_1000_run_149_f | 149 | 8 | -1704.2 | -1538.5 | 331.5 |
| Resultsadmix_corel_500_1000_run_150_f | 150 | 8 | -1698.3 | -1536.9 | 323.0 |
| Resultsadmix_corel_500_1000_run_151_f | 151 | 8 | -1702.3 | -1529.0 | 346.6 |
| Resultsadmix_corel_500_1000_run_152_f | 152 | 8 | -1702.2 | -1530.9 | 342.8 |
| Resultsadmix_corel_500_1000_run_153_f | 153 | 8 | -1696.3 | -1537.9 | 316.8 |
| Resultsadmix_corel_500_1000_run_154_f | 154 | 8 | -1704.1 | -1530.1 | 348.1 |
| Resultsadmix_corel_500_1000_run_155_f | 155 | 8 | -1700.3 | -1529.6 | 341.3 |
| Resultsadmix_corel_500_1000_run_156_f | 156 | 8 | -1698.0 | -1529.0 | 337.9 |
| Resultsadmix_corel_500_1000_run_157_f | 157 | 8 | -1709.7 | -1540.1 | 339.2 |
| Resultsadmix_corel_500_1000_run_158_f | 158 | 8 | -1699.5 | -1530.7 | 337.6 |
| Resultsadmix_corel_500_1000_run_159_f | 159 | 8 | -1987.0 | -1554.9 | 864.2 |
| Resultsadmix_corel_500_1000_run_160_f | 160 | 8 | -1693.6 | -1541.7 | 303.7 |
| Resultsadmix_corel_500_1000_run_161_f | 161 | 9 | -1633.0 | -1460.6 | 344.8 |
| Resultsadmix_corel_500_1000_run_162_f | 162 | 9 | -1632.7 | -1459.7 | 345.9 |
| Resultsadmix_corel_500_1000_run_163_f | 163 | 9 | -1637.1 | -1460.3 | 353.7 |
| Resultsadmix_corel_500_1000_run_164_f | 164 | 9 | -1639.6 | -1478.8 | 321.7 |
| Resultsadmix_corel_500_1000_run_165_f | 165 | 9 | -1636.0 | -1460.3 | 351.4 |
| Resultsadmix_corel_500_1000_run_166_f | 166 | 9 | -1648.8 | -1461.6 | 374.3 |
| Resultsadmix_corel_500_1000_run_167_f | 167 | 9 | -1632.0 | -1469.2 | 325.5 |
| Resultsadmix_corel_500_1000_run_168_f | 168 | 9 | -1637.8 | -1463.4 | 348.7 |
| Resultsadmix_corel_500_1000_run_169_f | 169 | 9 | -1640.7 | -1463.2 | 355.0 |
| Resultsadmix_corel_500_1000_run_170_f | 170 | 9 | -1635.4 | -1461.8 | 347.2 |
| Resultsadmix_corel_500_1000_run_171_f | 171 | 9 | -1633.9 | -1469.2 | 329.4 |
| Resultsadmix_corel_500_1000_run_172_f | 172 | 9 | -1633.9 | -1460.9 | 345.9 |
| Resultsadmix_corel_500_1000_run_173_f | 173 | 9 | -1650.2 | -1469.5 | 361.3 |
| Resultsadmix_corel_500_1000_run_174_f | 174 | 9 | -1634.4 | -1476.8 | 315.3 |
| Resultsadmix_corel_500_1000_run_175_f | 175 | 9 | -1631.4 | -1468.7 | 325.4 |
| Resultsadmix_corel_500_1000_run_176_f | 176 | 9 | -1633.2 | -1468.4 | 329.4 |
| Resultsadmix_corel_500_1000_run_177_f | 177 | 9 | -1630.6 | -1459.1 | 342.8 |
| Resultsadmix_corel_500_1000_run_178_f | 178 | 9 | -1630.9 | -1468.9 | 324.0 |
| Resultsadmix_corel_500_1000_run_179_f | 179 | 9 | -1637.8 | -1469.9 | 335.9 |
| Resultsadmix_corel_500_1000_run_180_f | 180 | 9 | -1641.2 | -1460.8 | 360.7 |
| Resultsadmix_corel_500_1000_run_181_f | 181 | 10 | -1571.2 | -1400.0 | 342.4 |
| Resultsadmix_corel_500_1000_run_182_f | 182 | 10 | -1568.4 | -1408.0 | 320.9 |
| Resultsadmix_corel_500_1000_run_183_f | 183 | 10 | -1584.2 | -1410.5 | 347.3 |
| Resultsadmix_corel_500_1000_run_184_f | 184 | 10 | -1574.0 | -1400.5 | 347.1 |
| Resultsadmix_corel_500_1000_run_185_f | 185 | 10 | -1582.6 | -1404.2 | 356.8 |
| Resultsadmix_corel_500_1000_run_186_f | 186 | 10 | -1569.8 | -1403.9 | 331.9 |
| Resultsadmix_corel_500_1000_run_187_f | 187 | 10 | -1583.4 | -1403.5 | 359.7 |
| Resultsadmix_corel_500_1000_run_188_f | 188 | 10 | -1582.3 | -1405.3 | 354.1 |
| Resultsadmix_corel_500_1000_run_189_f | 189 | 10 | -1580.4 | -1401.2 | 358.5 |
| Resultsadmix_corel_500_1000_run_190_f | 190 | 10 | -1575.1 | -1408.8 | 332.5 |
| Resultsadmix_corel_500_1000_run_191_f | 191 | 10 | -1571.2 | -1408.4 | 325.5 |
| Resultsadmix_corel_500_1000_run_192_f | 192 | 10 | -1571.1 | -1408.6 | 325.1 |
| Resultsadmix_corel_500_1000_run_193_f | 193 | 10 | -1573.9 | -1409.8 | 328.1 |
| Resultsadmix_corel_500_1000_run_194_f | 194 | 10 | -1569.3 | -1401.6 | 335.4 |
| Resultsadmix_corel_500_1000_run_195_f | 195 | 10 | -1578.6 | -1401.9 | 353.5 |
| Resultsadmix_corel_500_1000_run_196_f | 196 | 10 | -1575.1 | -1401.7 | 346.8 |
| Resultsadmix_corel_500_1000_run_197_f | 197 | 10 | -1576.6 | -1402.6 | 348.0 |
| Resultsadmix_corel_500_1000_run_198_f | 198 | 10 | -1572.5 | -1408.3 | 328.3 |
| Resultsadmix_corel_500_1000_run_199_f | 199 | 10 | -1577.5 | -1411.1 | 332.7 |
| Resultsadmix_corel_500_1000_run_200_f | 200 | 10 | -1587.8 | -1404.7 | 366.2 |

Result_deltaK_STRUCTUREHarvester


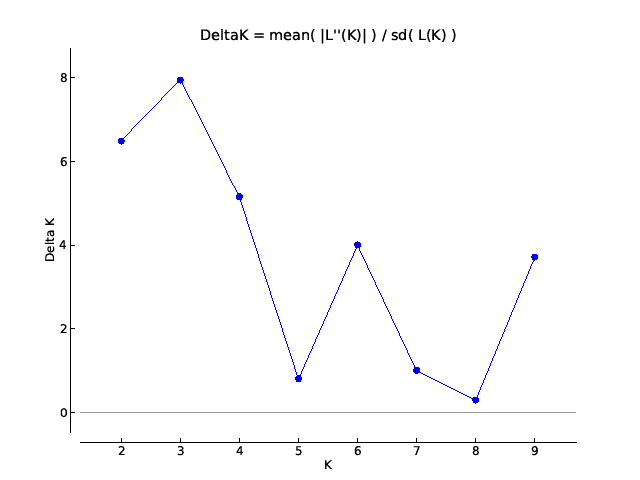


***Geneland Analysis***

The Geneland clustering model incorporates spatial information of the sampled individuals in addition to genetic diversity data, which may be preferable, since the assumption of spatial randomness for genetic structuring (which underlies Structure) is not necessarily meaningful in a biological manner (Guillot et al. 2009). Furthermore, Geneland allows accounting for spatial aspects like barriers to gene-flow, which may frequently occur (reviewed in Rieseberg et al. 2009). Geneland clusters individuals of different populations in such a way, that it maximizes Hardy-Weinberg equilibrium and linkage equilibrium for each cluster. The main advantage against other spatial clustering algorithms is that Geneland is based on a free Voronoi tessellation (for a discussion see Guillot et al. 2009) making the underlying spatial domain used for analysis independent from the sampling sites. A recent comparison shows that Geneland often outperforms other spatial clustering models frequently used for analyzing genetic data. This especially true when barriers were permeable to a certain degree, which is usual the case in empirical studies (Safner et al. 2011). Also for Geneland the correct estimation of the number of panmictic clusters (*K*) is important (Guillot et al. 2009), especially when isolation-by-distance (IBD) becomes a prominent aspect in the dataset (e.g. Frantz et al. 2009). Due to a lack of available methods that account for this issue, results have to be checked for IBD patterns and each cluster therein carefully for its biological relevance (Guillot et al. 2009). Initially, we calculated 25 independent chains with 3,000,000 generations each, to infer *K*, where *K* could vary between 1 and 10. For analysis, we sampled every 100^th^ generation and discarded the first 10% of the values as burn-in. Afterwards, we run additional 25 chains but with *K* fixed for the most frequent estimated *K* from the initial run using the same chain settings as mentioned above. From this, the run with the maximum average log posterior probability after burn-in was used (supplementary fig. S1, supplementary table S4).

For both clustering approaches, geographical distributions of genetic clusters were plotted using Diva-gis 7.5 (Hijmans et al. 2012) (not shown).

***Geographic information system (GIS)-based topographic maps***

ArcGIS 10 software from ESRI was used to draw the topographic maps. The GTOPO30 global digital elevation dataset developed by USGS was used as a basemap (http://eros.usgs.gov/#/Find_Data/Products_and_Data_Available/gtopo30_info). GPS coordinates for ‘wild *H. spontaneum*’ materials are given (supplementary table S1).

***Climate Data***

For species distribution modeling (SDM), we obtained averaged monthly climate data for present conditions from www.worldclim.org (vers. 1.4, Hijmans et al. 2005). Out of 19 bioclimatic relevant variables we selected a subset (BIO2, 7, 8, 9, 10, 11, 16, 17, 18, 19; for definitions see supplementary table S11), which we assume to be most relevant for our study species and which have a low degree of co-linearity (i.e. R^2^ < 0.75 tested via pairwise Pearson correlation coefficients). These variables were also compiled for the last glacial maximum (LGM) 21,000 years before present (yBP) from the worldclim homepage as well as computed for the mid Holocene climate optimum (MH) 6000 yBP following previous suggestions (Peterson and Nyári 2008; Ramirez and Jarvis 2008). To account for possible uncertainties caused by different global circulation models, we used two of them: MIROC (Hasumi and Emori 2004) and CCSM (Otto-Bliesner et al. 2006) available through the Paleoclimate Modelling Intercomparison Project Phase II (PMIP2; http://pmip2.lsce.ipsl.fr/, Braconnot et al. 2007a, b). Both paleoclimatic scenarios have been frequently applied to reconstruct historic potential distributions and are described in more detail in e.g. Waltari et al. (2007).

***Species Distribution Modeling***

A set of occurrence data from 360-point localities for subsp. *spontaneum* was compiled using data from different data sources: I) of self-collected material, II) germplasm repositories, III) herbarium information, IV) Global Biodiversity Information Facility (GBIF, www.gbif.org), V) Volis et al. (2001), VI) Tanyolac (2003), VII) the Flora of Turkey (Davis 1985), and VIII) the Flora Iranica (Rechinger 1970). In those cases where no coordinates were provided, the point localities were geo-referenced using the global gazetteer version 2.1 (www.fallingrain.com/world/) and the Britannica Atlas (Cleveland et al. 1994). These point localities cover the entire natural distribution area of subsp. *spontaneum*. Bothmer et al. (1989) reported the natural occurrence of subsp. *spontaneum*, as far as it can be traced at present, to reach from Greece, Egypt, and SW Asia through Iran, Afghanistan, to western Pakistan and southern Tajikistan. However, Harlan and Zohary (1966) and Zohary and Hopf (2000) argue against a natural distribution in western Turkey, Greece, Morocco, Libya, Egypt, Ethiopia, Tibet and China - thus for the modeling procedure we omitted sampling locations in these areas.

To account for possible uncertainties when using single algorithms for SDM development, we used an ensemble model approach as proposed by Araújo and New (2007) as implemented in Biomod vers. 1.1-5 (Thuiller 2003; Thuiller et al. 2009) for Cran R vers. 2.13.2 (R Development Core Team 2010). Biomod allows combining the strengths of different algorithms into well-defined ensemble prediction maps. Eight algorithms available in Biomod were used for modeling applying their standard settings. The complete set of algorithms comprised generalized linear models (GLM), generalized additive models (GAM), generalized boosting models (GBM), classification tree analysis (CTA), random forest (RF), multivariate adaptive regression splines (MARS), artificial neural networks (ANN), surface range envelopes (SRE) and flexible discriminant analysis (FDA).

As reliable absence data is commonly very difficult to compile, we used a set of pseudo-absence records to characterize the general environmental space available for the species. Therefore, we created as set of 5000 random background points within a 200 km buffer around the presence localities. This procedure accounts for dispersal limitations and thus represents a biological more meaningful scenario rather than a sampling across a broad unspecified area (Phillips et al. 2009; Vanderwal et al. 2009; Mateo et al. 2010). To assess the predictive ability of the different algorithms, ten evaluation runs were processed for each of them, wherein in each run 30% of the species records were randomly omitted from model training and used to test the model’s performance. Three different performance measures were used, i.e. the area under the receiver operating characteristic curve (AUC; Swets 1988), Cohen’s Kappa (Monserud and Leemans 1992) as well as the true skill statistic (TSS; Allouche et al. 2006). Model fit classifications for AUC and Kappa follow the respective classification schemes provided by Monserud and Leemans (1992) and Landis and Koch (1977) The relative importance of the bioclimatic variables in each single model was assessed using a permutation approach implemented in Biomod, which enables comparisons of variable contributions even between algorithms (see Thuiller et al. 2009 for further information).

The SDM computed under present climate conditions was subsequently projected onto the MH and LGM conditions and majority voting maps were produced based on binary presence-absence maps of each of the eight algorithms. Areas in which the environmental conditions exceed those present under current conditions in the training of the SDMs were quantified using multivariate environmental similarity surfaces (MESS), as proposed by Elith et al. (2010). Projecting SDMs in these areas requires extrapolation beyond their training range and is associated with an increased uncertainty (supplementary fig. S3).

**Literature Cited**

Allouche O, Tsoa A, Kadmon A. 2006. Assessing the accuracy of species distribution models: prevalence, kappa and the true skill statistic (TSS). J Appl Ecol. 43:1223-1232.

Araújo MB, New M. 2007. Ensemble forecasting of species distributions. Trends Ecol Evol. 22:42-47.

Bothmer R von, Yen C, Yang J. 1989. Does wild, six-rowed barley, *Hordeum agriocrithon*, really exist? Pl Genet Res Newsl. 77:17-19.

Braconnot P. et al. 2007a. Results of PMIP2 coupled simulations of the Mid-Holocene and Last Glacial Maximum – Part 1: experiments and large-scale features. Climate Past. 3:261–277.

Braconnot P. et al. 2007b. Results of PMIP2 coupled simulations of the Mid-Holocene and Last Glacial Maximum – Part 2: feedbacks with emphasis on the location of the ITCZ and mid- and high latitudes heat budget. Climate Past. 3:279-296.

Cleveland WA. et al. 1994. Britannica Atlas. Chicago Encyclopedia Britannica Inc.

Davis PH (ed) 1985. Flora of Turkey and the East Aegean Islands. Vol. 9. Edinburgh: University Press.

Earl DA, von Holdt BM. 2012. STRUCTURE HARVESTER: a website and program for visualizing STRUCTURE output and implementing the Evanno method. Conserv Genet Res. 4:359-361.

Elith J, Kearney M, Phillips S. 2010. The art of modeling range-shifting species. Methods Ecol Evol. 1:330-342.

Falush D, Stephens M, Pritchard JK. 2003. Inference of population structure using multilocus genotype data: linked loci and correlated allele frequencies. Genetics. 164:1567–1587.

Frantz AC. et al. 2009. Using spatial Bayesian methods to determine the genetic structure of a continuously distributed population: clusters or isolation by distance? J Appl Ecol. 46:493-505.

Guillot G. et al. 2009. Statistical methods in spatial genetics. Mol Ecol. 18:4734-4756.

Hall TA. 1999. BioEdit: a user-friendly biological sequence alignment editor and analysis program for Windows 95/98/NT. Nucleic Acids Symp Series. 41:95–98.

Harlan JR, Zohary D. 1966. Distribution of wild wheats and barley. Science. 153:1074–1080.

Hasumi H, Emori S, editors. 2004. K-1 Coupled GCM (MIROC) Description. K-1 Technical Report No. 1. Center for Climate System Research, University of Tokyo, Japan.

Hemming MN. et al. 2008. Low-temperature and day length cues are integrated to regulate *FLOWERING LOCUS T* in barley. Plant Physiol. 147:355–366.

Hijmans RJ. et al. 2005. Very high resolution interpolated climate surfaces for global land areas. Int J Climatol. 25:1965-1978.

Hijmans RJ, Guarino L, Mathur P. 2012. DIVA-GIS Version 7.5 Manual. http://wwww.diva-gis.org/docs/DIVA-GIS_manual_7.pdf.

Huson DH, Bryant D. 2006. Application of phylogenetic networks in evolutionary studies. Mol Biol Evol. 23:254–267.

Jakobsson M, Rosenberg NA. 2007. CLUMPP: a cluster matching and permutation program for dealing with label switching and multimodality in analysis of population structure. Bioinformatics. 23:1801-1806.

Kilian B. et al. 2006. Haplotype structure at seven barley genes: relevance to genepool bottlenecks, phylogeny of ear type and site of barley domestication. Mol Genet Genom. 276:230-241.

Landis JR, Koch GG. 1977. The measurement of observer agreement for categorical data. Biometrics. 33:159-174.

Mateo RG. et al. 2010. Profile or group discriminative techniques? Generating reliable species distribution models using pseudo-absences and target-group absences from natural history collections.” Divers Distrib. 16:84-94.

Monserud RA, Leemans R. 1992. Comparing global vegetation maps with Kappa statistics. Ecol Modell. 62:275-293.

Morrell PL, Lundy KE, Clegg MT. 2003. Distinct geographic patterns of genetic diversity are maintained in wild barley (*Hordeum vulgare* ssp. *spontaneum*) despite migration. Proc Nat Acad Sci USA. 100:10812-10817.

Otto-Bliesner BL. et al. 2006. Last Glacial Maximum and Holocene climate in CCSM3. J Climate. 19:2526–2544.

Peterson AT, Nyári AS. 2008. Ecological niche conservatism and Pleistocene refugia in the Thrush-like Mourner, *Shiffornis* sp., in the Neotropics. Evolution. 62:173-183.

Phillips SJ. et al. 2009. Sample selection bias and presence-only distribution models: implications for background and pseudo-absence data. Ecol Appl. 19:181-197.

Pritchard JK, Stephens M, Donnelly P. 2000. Inference of population structure using multilocus genotype data. Genetics. 155:945–959.

Pritchard JK, Wen W. 2004. Documentation for structure software: version 2. http://pritch.bsd.uchicago.edu/software/readme_structure2_1.pdf.

Ramirez J, Jarvis A. 2008. High Resolution Statistically Downscaled Future Climate Surfaces. International Centre for Tropical Agriculture, CIAT. http://gisweb.ciat.cgiar.org/GCMPage.

Rechinger KH. 1970. Flora Iranica. Gramineae. Graz: Akademische Druck- und Verlagsgesellschaft.

Rieseberg L, Vines T, Kane N. 2009. Editorial and Retrospective 2008. Mol Ecol. 18:1-20.

Rozen S, Skaletsky H. 2000. Primer3 on the WWW for general users and for biologist programmers. Methods Mol Biol. 132:365-386.

Safner T. et al. 2011. Comparison of Bayesian clustering and edge detection methods for inferring boundaries in landscape genetics. Int J Mol Sci. 12:865-889.

Swets K. 1988. Measuring the accuracy of diagnostic systems. Science. 240:1285-1293.

Tanyolac B. 2003 [Inter-simple sequence repeat (ISSR) and RAPD variation among wild barley *(Hordeum. vulgare* subsp. *spontaneum*) populations from west Turkey](http://www.springerlink.com/content/uu715r4221560j21/). [Genet Res Crop Evol](http://www.springerlink.com/content/0925-9864/). 50:611-614.

Thuiller W. 2003. BIOMOD - optimizing predictions of species distributions and projecting potential future shifts under global change. Global Change Biol. 9:1352-1362.

Thuiller W. et al. 2009. BIOMOD - a platform for ensemble forecasting of species distributions. Ecography. 32:369-373.

Turner A. et al. 2005. The pseudo-response regulator *Ppd-H1* provides adaptation to photoperiod in barley. Science. 310:1031–1034.

Vanderwal J. et al. 2009. Selecting pseudo-absence data for presence-only distribution modeling: How far should you stray from what you know? Ecol Modelling 220:589-594.

Volis S. et al. 2001. Allozyme variation in Turkmenian populations of wild barley, *Hordeum spontaneum* Koch. Ann Bot. 87:435-446.

Waltari E. et al. 2007. Locating Pleistocene Refugia: Comparing Phylogeographic and Ecological Niche Model Predictions. PLoS ONE. 2(7):e563. doi:10.1371/journal.pone.0000563.

Zohary D, Hopf M. 2000. Domestication of Plants in the Old World. 3rd edition. Oxford: Oxford University Press.

Supplementary figures


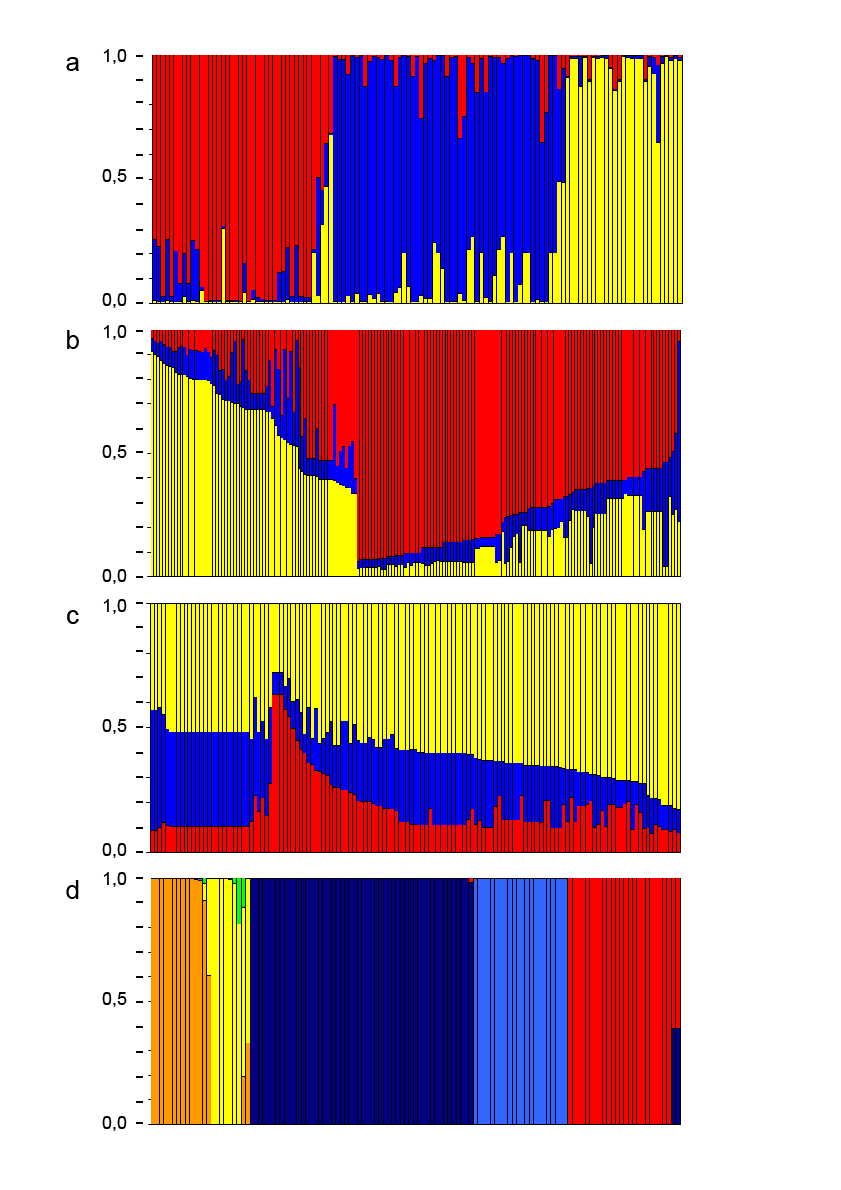


(*previous page*): **Supplementary figure S1** Cluster assignments of 415 genotypes resequenced at seven gene loci. **a)** Assignments of 123 genotypes of ‘wild *H. spontaneum*’ to three clusters based on Structure (*K*=3). X-axis: individual genotypes; Y-axis: proportion of membership (%); Yellow, ‘Western Cluster’; Blue, ‘Turkish Cluster’; Red, ‘Eastern Cluster.’

**b)** Assignments of 152 genotypes of ‘genebank *H. spontaneum*’ to the three Structure (*K*=3) inferred clusters of ‘wild *H. spontaneum*’. Abbreviations and notes as in **a**. **c)** Assignments of 140 genotypes of *Hordeum vulgare* (domesticated barley) to the three Structure (*K*=3) inferred clusters of ‘wild *H. spontaneum*’. Abbreviations and notes as in **a**.

**d)** Assignments of 123 genotypes of ‘wild *H. spontaneum*’ to five clusters based on Geneland (*K*=5). X-axis: genotypes; Y-axis: proportion of membership (%); Orange, ‘Northern (N) Levant Cluster’; Yellow, ‘Southern (S) Levant Cluster’; Dark blue, ‘Southeastern (SE) Turkish Cluster’; Light blue, Southern (S) Turkish Cluster’; Red, ‘Eastern Cluster’ and green, ‘ghost cluster’. X-axis: genotypes; Y-axis: proportion of membership (%).

**
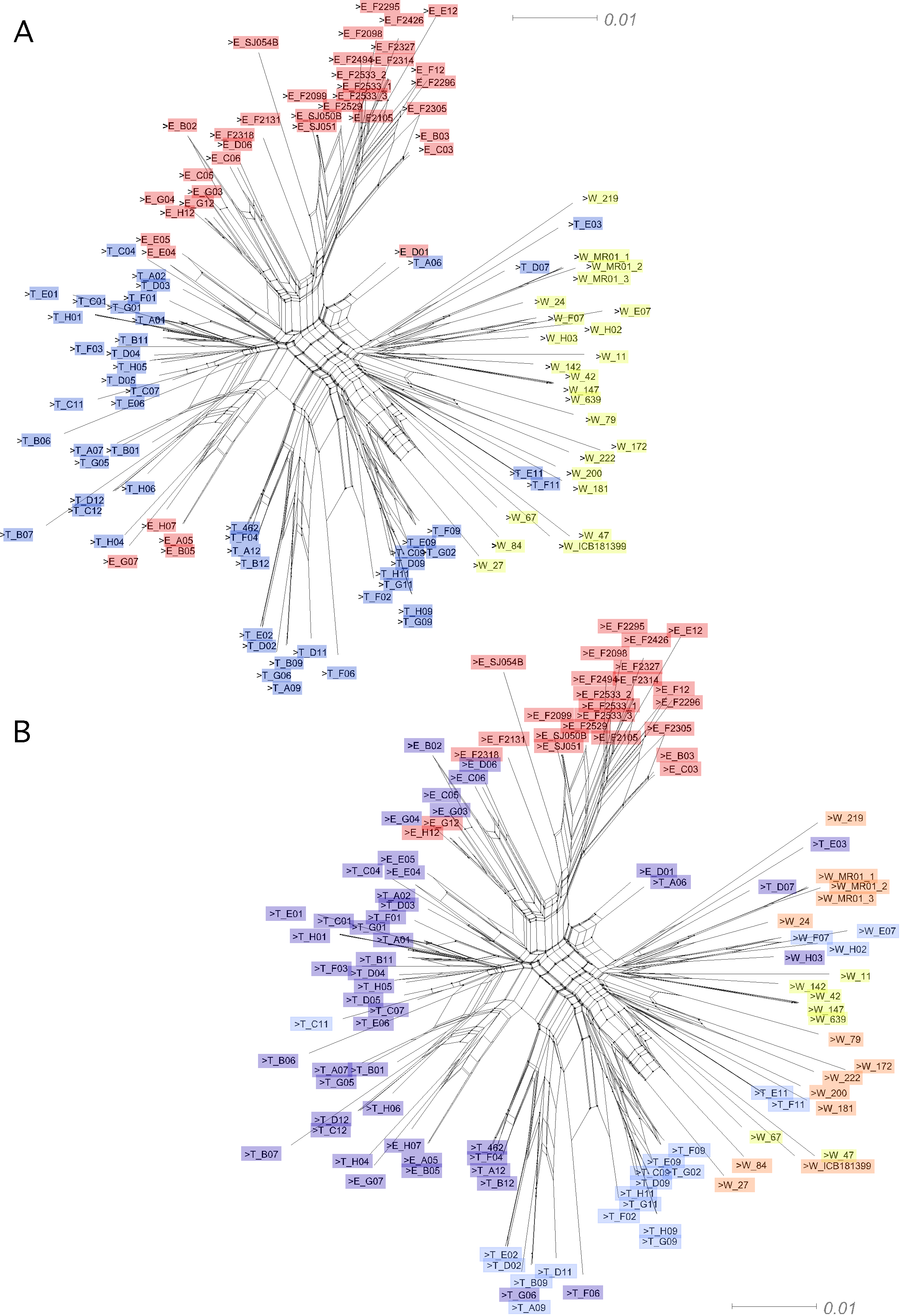
**

(*previous page*): **Supplementary figure S2** NeighborNet planar graph of uncorrected P distances (Hamming distances) for 183 characters among 114 ‘wild *H. spontaneum*’ genotypes. (A) Individuals were highlighted based on Structure (*K* = 3)-inferred clusters as in Fig.2a. (B) Individuals were highlighted based on Geneland (*K* = 5)-inferred clusters as in Fig.2b. Admixed individuals based on Structure were not considered. A matrix was created of one row for each individual line and two columns for each of two possible alleles at each locus into which the haplotypes were written, coded as single ASCII characters each.

Accession numbers as in supplementary table 1.


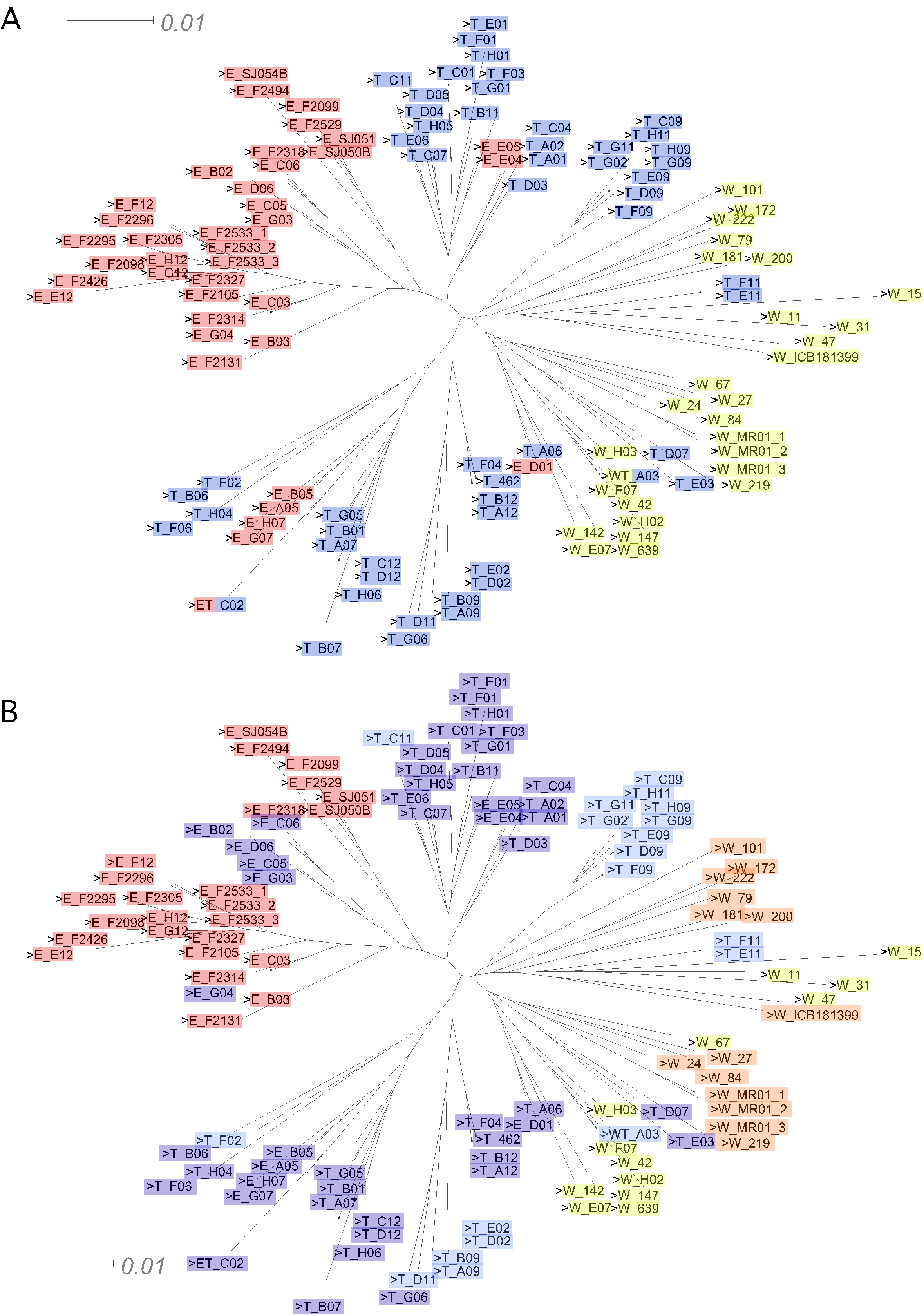


(*previous page*): **Supplementary figure S3** Neighbor-joining (NJ) strict consensus tree of uncorrected P distances (Hamming distances) for 183 characters among 119 ‘wild *H. spontaneum*’ genotypes. (A) Individuals were highlighted based on Structure (*K* = 3)-inferred clusters as in Fig.2a. (B) Individuals were highlighted based on Geneland (*K* = 5)-inferred clusters as in Fig.2b. Accession numbers as in supplementary table 1.


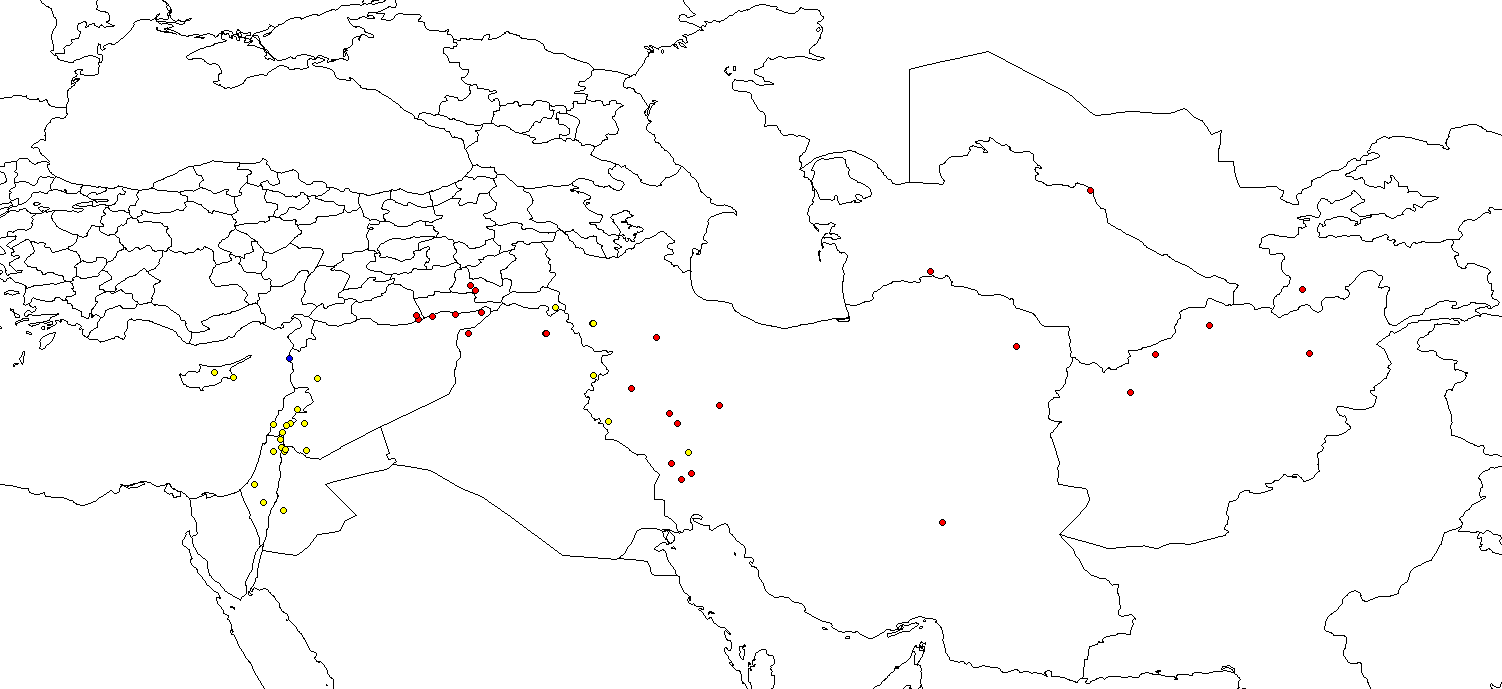


**Supplementary figure S4** Potential collection sites of 152 ‘genebank *H. spontaneum*’ genotypes and their assignment to the ‘wild *H. spontaneum*’ clusters inferred by Structure (*K*=3). Yellow circles, ‘Western Cluster’; Blue circle, ‘Turkish Cluster’; Red, circles ‘Eastern Cluster’.

**
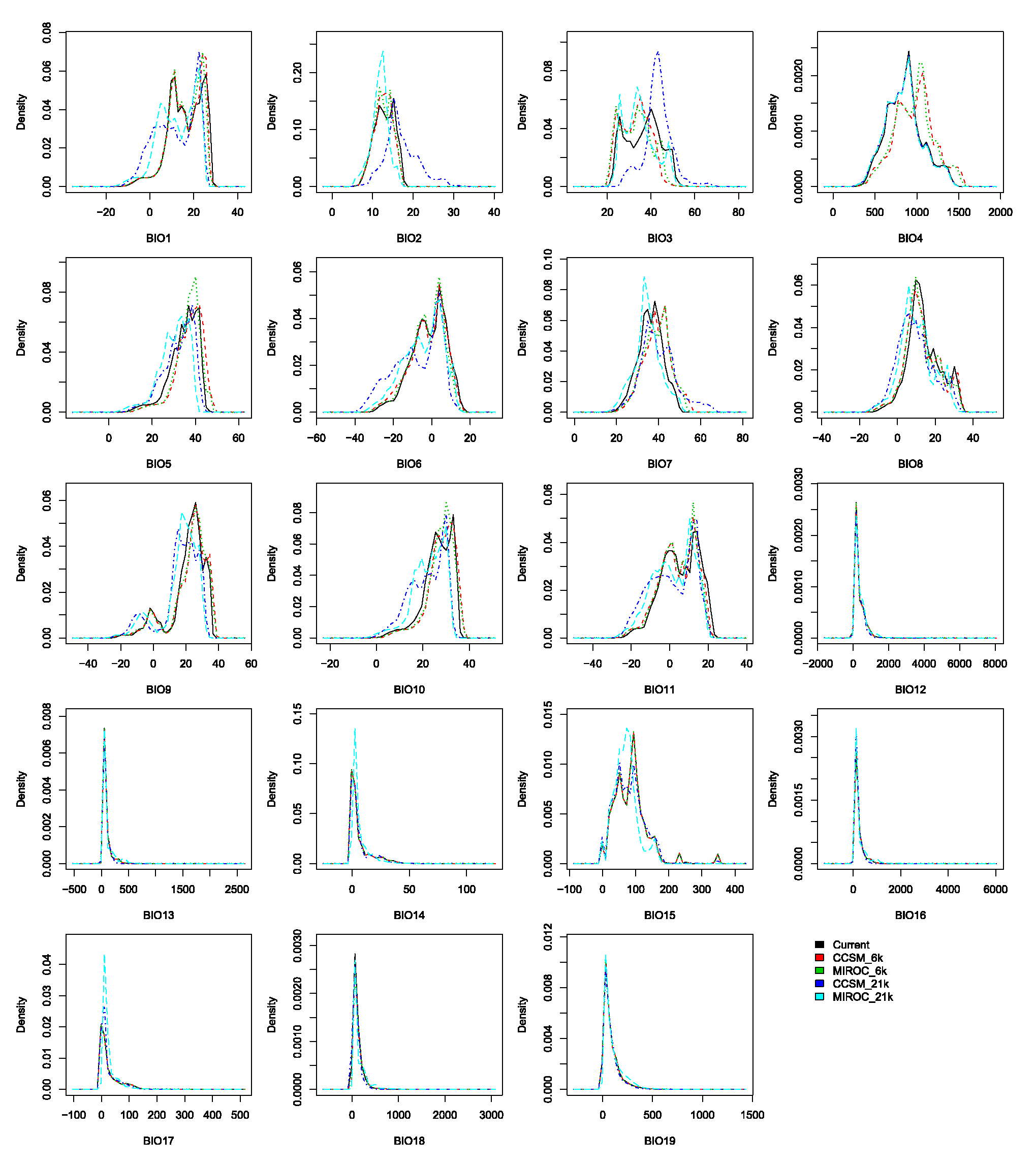
**

**Supplementary figure S5** Comparison of 19 bioclimatic variables used in two global circulation models (MIROC, CCSM) to model the potential current and paleodistributions 6,000 and 21,000 years ago.

Supplementary tables

**Supplementary table S1** Details for all 415 genotypes resequenced at seven loci. All available information is provided for accession numbers, collectors, seed sources and geographical origins.

Table provided as separately spread sheet.

**Supplementary table S2** Further details for all 415 genotypes resequenced at seven loci. Haplotype numbers (two alleles considered) are provided. Missing information is highlighted in yellow, heterozygous loci are highlighted in brown.

Table provided as separately spread sheet.

**Supplementary table S3** Further details for all 415 genotypes resequenced at seven loci. Haplotype numbers and frequencies (%) are provided for Structure inferred groups (*K* = 3). The data are shown for three groups of ‘wild *H. spontaneum*’, ‘genebank *H. spontaneum*’ and domesticated barley accessions. West_wild - ‘Western Cluster’; Turkey_wild - ‘Turkish Cluster’; East_wild - ‘Eastern Cluster’; West_Genebank - genebank accessions assigned to the wild ‘Western Cluster’; Turkey_Genebank - genebank accessions assigned to the wild ‘Western Cluster’; East_Genebank - genebank accessions assigned to the wild ‘Western Cluster’; Ns, not significant.

Table provided as separately spread sheet.

**Supplementary table S4** Further details for all 415 genotypes resequenced at seven loci. Assignments (%) for 123 wild barley genotypes (‘wild *H. spontaneum*’) are shown for i) Structure inferred groups (*K* = 3), ii) Geneland inferred groups (*K* = 6) and NNet clusters. Assignments (%) to Structure inferred groups (*K* = 3) are presented for 152 genotypes of ‘genebank *H. spontaneum’* and 140 domesticated barleys (landraces, cultivars and *H. agriocrithon*). Outliers and hybrids are indicated.

Table provided as separately spread sheet.

**Supplementary table S5** Harmonic means of Dest across seven loci (Josts' D 2008) for Structure inferred groups (*K* = 3) of ‘wild *H. spontaneum*’ and *H. vulgare* are shown above the diagonal, FST values (FSTAT) are presented below the diagonal (95% CI).

|  | | |  |  |
| --- | --- | --- | --- | --- |
|  |  |  |  |  |
|  | East | Turkey | West | *H. vulgare* |
| East | -- | 0,31 | 0,28 | 0,45 |
| Turkey | 0.26 ** (0.16-0.38) | -- | 0,22 | 0,25 |
| West | 0.24 ** (0.09-0.41) | 0.15 ** (0.07-0.23) | -- | 0,33 |
| *H. vulgare* | 0.32 ** (0.24-0.43) | 0.22 ** (0.11-0.34) | 0.18 ** (0.11-0.26) | -- |

**Supplementary table S6** Harmonic means of Dest across the seven loci (Josts' D 2008) for Geneland inferred groups (*K* = 5) of ‘wild *H. spontaneum*’ and *H. vulgare* are shown above the diagonal, FST values (FSTAT) are presented below the diagonal (95% CI).

|  | | |  |  |  |
| --- | --- | --- | --- | --- | --- |
|  |  |  |  |  |  |
|  | East | N-Levant | S-Levant | S-Turkey | SE-Turkey |
| East | -- | 0,44 | 0,24 | 0,54 | 0,24 |
| N-Levant | 0.37 *** (0.17-0.56) | -- | 0,24 | 0,22 | 0,26 |
| S-Levant | 0.33 *** (0.11-0.53) | 0.11 *** (0.04-0.19) | -- | 0,41 | 0,31 |
| S-Turkey | 0.44 *** (0.30-0.59) | 0.14 *** (0.08-0.18) | 0.26 *** (0.16-0.39) | -- | 0,19 |
| SE-Turkey | 0.29 *** (0.13-0.51) | 0.18 *** (0.06-0.33) | 0.23 *** (0.1-0.36) | 0.2 *** (0.06-.033) | -- |

**Supplementary table S7** Genetic diversity at seven loci. Values for R (n=10) and Nei’s gene diversity (Nei 1987, FSTAT) are given for Structure inferred groups (*K* = 3).


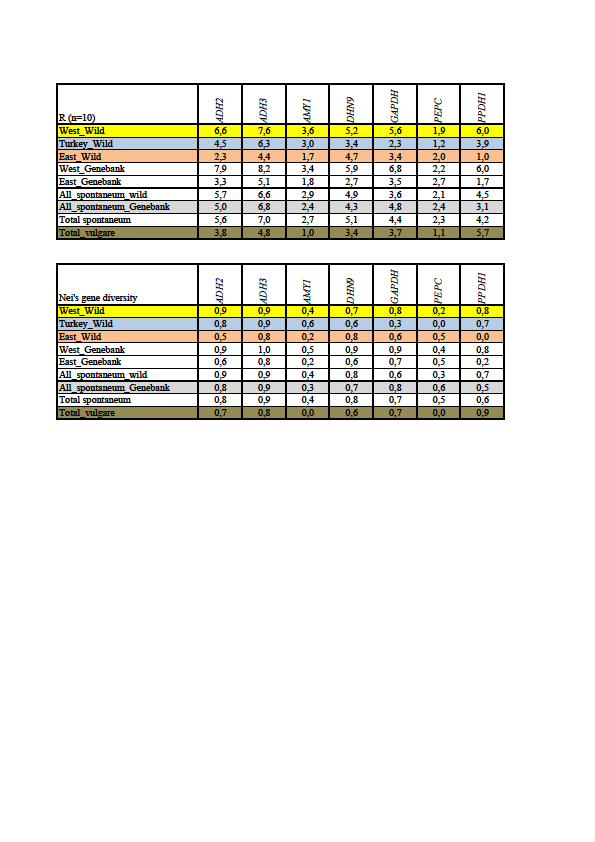


**Supplementary table S8** Genetic diversity at seven loci. Values for R (n=5) and Nei’s gene diversity (Nei 1987, FSTAT) are given for wild barleys (‘wild *H. spontaneum*’) for Geneland inferred groups (*K* = 5). S-Levant - ‘Southern (S) Levant Cluster’; N-Levant - ‘Northern (N) Levant Cluster’; S-Turkey - ‘Southern (S) Turkish Cluster’; SE-Turkey - ‘Southeastern (SE) Turkish Cluster’; East - ‘Eastern Cluster’.

|  | | |  |  |  |  |  |
| --- | --- | --- | --- | --- | --- | --- | --- |
|  |  |  |  |  |  |  |  |
| **R (n=5)** | *ADH2* | *ADH3* | *AMY1* | *DHN9* | *GAPDH* | *PEPC* | *PPDH1* |
| S-Levant | 3,5 | 3,8 | 2,0 | 3,2 | 2,5 | 2,3 | 3,7 |
| N-Levant | 3,8 | 4,3 | 3,0 | 3,8 | 3,4 | 1,0 | 3,0 |
| S-Turkey | 3,3 | 3,1 | 1,8 | 2,7 | 1,6 | 1,0 | 3,2 |
| SE-Turkey | 3,6 | 3,9 | 2,4 | 2,4 | 2,3 | 1,2 | 1,9 |
| East | 1,7 | 2,8 | 1,0 | 3,1 | 2,5 | 1,7 | 1,2 |
|  |  |  |  |  |  |  |  |
|  |  |  |  |  |  |  |  |
| **Nei's gene diversity** | *ADH2* | *ADH3* | *AMY1* | *DHN9* | *GAPDH* | *PEPC* | *PPDH1* |
| S-Levant | 0,806 | 0,819 | 0,319 | 0,722 | 0,639 | 0,464 | 0,861 |
| N-Levant | 0,857 | 0,923 | 0,615 | 0,819 | 0,791 | 0 | 0,736 |
| S-Turkey | 0,747 | 0,721 | 0,368 | 0,645 | 0,247 | 0 | 0,749 |
| SE-Turkey | 0,827 | 0,87 | 0,569 | 0,547 | 0,532 | 0,057 | 0,356 |
| East | 0,323 | 0,691 | 0 | 0,708 | 0,557 | 0,323 | 0,08 |

**Supplementary table S9.** Model performance in terms of AUC, Kappa and TSS. Abbreviations are: AUC – area under the receiver operating characteristic curve; GLM - generalized linear models; GAM - generalized additive models; GBM - generalized boosting models; CTA - classification tree analysis; RF - random forest; MARS - multivariate adaptive regression splines; ANN - artificial neural networks; SRE - surface range envelopes; FDA - flexible discriminant analysis; TSS – true skills statistic.

|  | | |  |  |  |  |  |
| --- | --- | --- | --- | --- | --- | --- | --- |
|  |  |  |  |  |  |  |  |
|  | AUC CV | Rank | Kappa CV | Rank | TSS CV | Rank | Mean rank |
| ANN | 0,91 | 5 | 0,539 | 4 | 0,707 | 6 | 5 |
| CTA | 0,857 | 7 | 0,472 | 6 | 0,616 | 8 | 7 |
| GAM | 0,936 | 3 | 0,513 | 3 | 0,747 | 4 | 3 |
| GBM | 0,95 | 2 | 0,578 | 2 | 0,783 | 2 | 2 |
| GLM | 0,936 | 3 | 0,513 | 3 | 0,754 | 3 | 3 |
| MARS | 0,924 | 4 | 0,485 | 5 | 0,717 | 5 | 5 |
| FDA | 0,902 | 6 | 0,471 | 7 | 0,68 | 7 | 7 |
| RF | 0,953 | 1 | 0,609 | 1 | 0,795 | 1 | 1 |
| SRE | NA | 8 | 0,369 | 8 | 0,611 | 9 | 8 |
| mean | 0,921 |  | 0,5225 |  | 0,724875 |  |  |

**Supplementary table S10.** Ensemble weights of the single algorithms in the final species distribution model. For abbreviations see supplementary table S9.

|  | |
| --- | --- |
|  |  |
|  | Weights |
| ANN | 0,0366 |
| CTA | 0,0143 |
| GAM | 0,1219 |
| GBM | 0,24 |
| GLM | 0,1219 |
| MARS | 0,0586 |
| FDA | 0,0229 |
| RF | 0,3839 |
| SRE | 0 |
|  | 1,0001 |

**Supplementary table S11.** Variable importance suggested by the single algorithms of species distribution model. For abbreviations see supplementary table S9.

|  | spon.BIO2 | spon.BIO7 | spon.BIO8 | spon.BIO9 | spon.BIO10 | spon.BIO11 | spon.BIO16 | spon.BIO17 | spon.BIO18 | spon.BIO19 |
| --- | --- | --- | --- | --- | --- | --- | --- | --- | --- | --- |
| ANN | 0,226 | 0,206 | 0,321 | 0,194 | 0,269 | 0,432 | 0,644 | 0,556 | 0,292 | 0,79 |
| CTA | 0,327 | 0,21 | 0,035 | 0,353 | 0,018 | 0,11 | 0,116 | 0,213 | 0,017 | 0,638 |
| GAM | 0,063 | 0,158 | 0,136 | 0,23 | 0,066 | 0,316 | 0,388 | 0,249 | 0,052 | 0,09 |
| GBM | 0,089 | 0,016 | 0,02 | 0,247 | 0,044 | 0,08 | 0,06 | 0,063 | 0,087 | 0,42 |
| GLM | 0,056 | 0,219 | 0,325 | 0,537 | 0,129 | 0,695 | 0,697 | 0,043 | 0,362 | 0,163 |
| MARS | 0,089 | 0,062 | 0 | 0,087 | 0 | 0,116 | 0 | 0,459 | 0 | 0,808 |
| FDA | 0,218 | 0,06 | 0 | 0,078 | 0,195 | 0,192 | 0,189 | 0,402 | 0 | 0,172 |
| RF | 0,116 | 0,06 | 0,055 | 0,264 | 0,073 | 0,08 | 0,178 | 0,08 | 0,147 | 0,314 |
| SRE | 0,037 | 0,022 | 0,04 | 0,018 | 0,021 | 0,034 | 0,036 | 0,007 | 0,001 | 0,089 |

| BIO1 = Annual Mean Temperature; |
| --- |
| BIO2 = Mean Diurnal Range (Mean of monthly (max temp - min temp)) |
| BIO3 = Isothermality (BIO2/BIO7) (* 100) |
| BIO4 = Temperature Seasonality (standard deviation *100) |
| BIO5 = Max Temperature of Warmest Month |
| BIO6 = Min Temperature of Coldest Month |
| BIO7 = Temperature Annual Range (BIO5-BIO6) |
| BIO8 = Mean Temperature of Wettest Quarter |
| BIO9 = Mean Temperature of Driest Quarter |
| BIO10 = Mean Temperature of Warmest Quarter |
| BIO11 = Mean Temperature of Coldest Quarter |
| BIO12 = Annual Precipitation |
| BIO13 = Precipitation of Wettest Month |
| BIO14 = Precipitation of Driest Month |
| BIO15 = Precipitation Seasonality (Coefficient of Variation) |
| BIO16 = Precipitation of Wettest Quarter |
| BIO17 = Precipitation of Driest Quarter |
| BIO18 = Precipitation of Warmest Quarter |
| BIO19 = Precipitation of Coldest Quarter |

**Supplementary table S12** GenBank accession numbers of 184 haplotypes obtained in this study (including haplotypes based on microsatellite differences). *Adh2* (30 haplotypes; incl. HT6 from accession number PI420911_EMBL); *Adh3*: (47 haplotypes; incl. HT36 from PI227019_235); *Amy1*: (12 haplotypes); *Dhn9*: (31 haplotypes; incl. HT27 from F2097 and HT31 from 270); *G3pdh*: (23 haplotypes; incl. HT15 from PI249983_236 and HT16 from PI254894_1; PI254894_2; PI254894_5; PI254894_637); *Pepc*: (8 haplotypes; incl. HT6 from F05); *Ppdh1*: (33 haplotypes; incl. HT8 from B04; HT22 from F2097 and HT60 from 590).

See supplementary table S2 for all haplotype numbers (two alleles considered) for all 415 genotypes resequenced at seven loci. Alignment length in base pairs (bp): *Adh2*: 1.017; *Adh3*: 829; *Amy1*: 650; *Dhn9*: 763; *Gapdh (G3pdh)*: 765; *Pepc*: 893; *Ppdh1*: 894. Thus c. 2.4 Mbp of sequence information is provided (c. 5.800 bp per genotype).

|  | | |
| --- | --- | --- |
| **Locus** | **Haplotype** | **Accession number** |
| ***Adh2*** | 1 | KC661080 |
|  | 2 | KC661081 |
|  | 3 | KC661082 |
|  | 4 | KC661083 |
|  | 5 | KC661084 |
|  | 6 | KC661085 |
|  | 7 | KC661086 |
|  | 8 | KC661087 |
|  | 9 | KC661088 |
|  | 10 | KC661089 |
|  | 11 | KC661090 |
|  | 12 | KC661091 |
|  | 13 | KC661092 |
|  | 14 | KC661093 |
|  | 15 | KC661094 |
|  | 16 | KC661095 |
|  | 17 | KC661096 |
|  | 18 | KC661097 |
|  | 20 | KC661098 |
|  | 21 | KC661099 |
|  | 22 | KC661100 |
|  | 23 | KC661101 |
|  | 24 | KC661102 |
|  | 25 | KC661103 |
|  | 26 | KC661104 |
|  | 27 | KC661105 |
|  | 28 | KC661106 |
|  | 29 | KC661107 |
|  | 30 | KC661108 |
|  | 32 | KC661109 |
| ***Adh3*** | 1 | KC661110 |
|  | 2 | KC661111 |
|  | 3 | KC661112 |
|  | 4 | KC661113 |
|  | 5 | KC661114 |
|  | 6 | KC661115 |
|  | 7 | KC661116 |
|  | 8 | KC661117 |
|  | 9 | KC661118 |
|  | 10 | KC661119 |
|  | 11 | KC661120 |
|  | 12 | KC661121 |
|  | 13 | KC661122 |
|  | 14 | KC661123 |
|  | 15 | KC661124 |
|  | 16 | KC661125 |
|  | 17 | KC661126 |
|  | 18 | KC661127 |
|  | 19 | KC661128 |
|  | 20 | KC661129 |
|  | 21 | KC661130 |
|  | 22 | KC661131 |
|  | 23 | KC661132 |
|  | 24 | KC661133 |
|  | 25 | KC661134 |
|  | 26 | KC661135 |
|  | 27 | KC661136 |
|  | 28 | KC661137 |
|  | 29 | KC661138 |
|  | 30 | KC661139 |
|  | 31 | KC661140 |
|  | 32 | KC661141 |
|  | 33 | KC661142 |
|  | 34 | KC661143 |
|  | 35 | KC661144 |
|  | 36 | KC661145 |
|  | 38 | KC661146 |
|  | 39 | KC661147 |
|  | 40 | KC661148 |
|  | 41 | KC661149 |
|  | 42 | KC661150 |
|  | 43 | KC661151 |
|  | 44 | KC661152 |
|  | 45 | KC661153 |
|  | 46 | KC661154 |
|  | 47 | KC661155 |
|  | 48 | KC661156 |
| ***Amy1*** | 1 | KC661157 |
|  | 2 | KC661158 |
|  | 3 | KC661159 |
|  | 4 | KC661160 |
|  | 5 | KC661161 |
|  | 6 | KC661162 |
|  | 7 | KC661163 |
|  | 8 | KC661164 |
|  | 9 | KC661165 |
|  | 10 | KC661166 |
|  | 11 | KC661167 |
|  | 12 | KC661168 |
| ***Dhn9*** | 1 | KC661169 |
|  | 2 | KC661170 |
|  | 3 | KC661171 |
|  | 5 | KC661172 |
|  | 6 | KC661173 |
|  | 7 | KC661174 |
|  | 8 | KC661175 |
|  | 9 | KC661176 |
|  | 11 | KC661177 |
|  | 13 | KC661178 |
|  | 14 | KC661179 |
|  | 15 | KC661180 |
|  | 16 | KC661181 |
|  | 17 | KC661182 |
|  | 18 | KC661183 |
|  | 20 | KC661184 |
|  | 21 | KC661185 |
|  | 23 | KC661186 |
|  | 24 | KC661187 |
|  | 25 | KC661188 |
|  | 26 | KC661189 |
|  | 27 | KC661190 |
|  | 28 | KC661191 |
|  | 29 | KC661192 |
|  | 30 | KC661193 |
|  | 31 | KC661194 |
|  | 32 | KC661195 |
|  | 33 | KC661196 |
|  | 35 | KC661197 |
|  | 36 | KC661198 |
|  | 37 | KC661199 |
| ***GAPDH*** | 1 | KC661200 |
|  | 2 | KC661201 |
|  | 3 | KC661202 |
|  | 4 | KC661203 |
|  | 5 | KC661204 |
|  | 6 | KC661205 |
|  | 7 | KC661206 |
|  | 8 | KC661207 |
|  | 9 | KC661208 |
|  | 10 | KC661209 |
|  | 11 | KC661210 |
|  | 12 | KC661211 |
|  | 13 | KC661212 |
|  | 15 | KC661213 |
|  | 16 | KC661214 |
|  | 17 | KC661215 |
|  | 18 | KC661216 |
|  | 19 | KC661217 |
|  | 20 | KC661218 |
|  | 21 | KC661219 |
|  | 22 | KC661220 |
|  | 23 | KC661221 |
|  | 27 | KC661222 |
| ***PEPC*** | 1 | KC661223 |
|  | 2 | KC661224 |
|  | 3 | KC661225 |
|  | 4 | KC661226 |
|  | 5 | KC661227 |
|  | 6 | KC661228 |
|  | 7 | KC661229 |
|  | 8 | KC661230 |
| ***PPD-H1*** | 1 | KC661231 |
|  | 2 | KC661232 |
|  | 3 | KC661233 |
|  | 4 | KC661234 |
|  | 5 | KC661235 |
|  | 7 | KC661236 |
|  | 8 | KC661237 |
|  | 9 | KC661238 |
|  | 11 | KC661239 |
|  | 16 | KC661240 |
|  | 17 | KC661241 |
|  | 18 | KC661242 |
|  | 19 | KC661243 |
|  | 21 | KC661244 |
|  | 22 | KC661245 |
|  | 24 | KC661246 |
|  | 25 | KC661247 |
|  | 28 | KC661248 |
|  | 31 | KC661249 |
|  | 34 | KC661250 |
|  | 40 | KC661251 |
|  | 41 | KC661252 |
|  | 46 | KC661253 |
|  | 51 | KC661254 |
|  | 52 | KC661255 |
|  | 53 | KC661256 |
|  | 54 | KC661257 |
|  | 55 | KC661258 |
|  | 56 | KC661259 |
|  | 57 | KC661260 |
|  | 58 | KC661261 |
|  | 59 | KC661262 |
|  | 60 | KC661263 |
